# Transcriptome and Metabolome Reprogramming in Tomato Plants by *Trichoderma harzianum strain* T22 Primes and Enhances Defense Responses Against Aphids

**DOI:** 10.3389/fphys.2019.00745

**Published:** 2019-06-21

**Authors:** Mariangela Coppola, Gianfranco Diretto, Maria Cristina Digilio, Sheridan Lois Woo, Giovanni Giuliano, Donata Molisso, Francesco Pennacchio, Matteo Lorito, Rosa Rao

**Affiliations:** ^1^Department of Agricultural Sciences, Portici, Italy; ^2^ENEA, Rome, Italy; ^3^Task Force on Microbiome Studies, University of Naples Federico II, Naples, Italy; ^4^Department of Pharmacy, University of Naples Federico II, Naples, Italy; ^5^National Research Council, Institute for Sustainable Plant Protection, Portici, Italy

**Keywords:** San Marzano, aphid, RNA-Seq, semi-polarmetabolome, defense

## Abstract

Beneficial fungi in the genus *Trichoderma* are among the most widespread biocontrol agents of plant pathogens. Their role in triggering plant defenses against pathogens has been intensely investigated, while, in contrast, very limited information is available on induced barriers active against insects. The growing experimental evidence on this latter topic looks promising, and paves the way toward the development of *Trichoderma* strains and/or consortia active against multiple targets. However, the predictability and reproducibility of the effects that these beneficial fungi is still somewhat limited by the lack of an in-depth understanding of the molecular mechanisms underlying the specificity of their interaction with different crop varieties, and on how the environmental factors modulate this interaction. To fill this research gap, here we studied the transcriptome changes in tomato plants (cultivar “Dwarf San Marzano”) induced by *Trichoderma harzianum* (strain T22) colonization and subsequent infestation by the aphid *Macrosiphum euphorbiae*. A wide transcriptome reprogramming, related to metabolic processes, regulation of gene expression and defense responses, was induced both by separate experimental treatments, which showed a synergistic interaction when concurrently applied. The most evident expression changes of defense genes were associated with the multitrophic interaction *Trichoderma*-tomato-aphid. Early and late genes involved in direct defense against insects were induced (i.e., *peroxidase, GST, kinases and polyphenol oxidase, miraculin, chitinase*), along with indirect defense genes, such as *sesquiterpene synthase* and *geranylgeranyl phosphate synthase*. Targeted and untargeted semi-polar metabolome analysis revealed a wide metabolome alteration showing an increased accumulation of isoprenoids in *Trichoderma* treated plants. The wide array of transcriptomic and metabolomics changes nicely fit with the higher mortality of aphids when feeding on *Trichoderma* treated plants, herein reported, and with the previously observed attractiveness of these latter toward the aphid parasitoid *Aphidius ervi*. Moreover, *Trichoderma* treated plants showed the over-expression of transcripts coding for several families of defense-related transcription factors (bZIP, MYB, NAC, AP2-ERF, WRKY), suggesting that the fungus contributes to the priming of plant responses against pest insects. Collectively, our data indicate that *Trichoderma* treatment of tomato plants induces transcriptomic and metabolomic changes, which underpin both direct and indirect defense responses.

## Introduction

*Solanum lycopersicum* represents one of the most widespread horticultural crops in the world, with a production of 177 million of tons in 2016 (FAOSTAT). Pests and pathogens cause remarkable crop losses only in part limited by control strategies, which are still largely based on chemical pesticides. The use of biocontrol agents and/or the implementation of bioinspired strategies of sustainable pest management (Pennacchio et al., 2012) is still limited, in spite of the health and environmental issues associated with pesticide release (Alewu and Nosiri, 2011) and the recent changes of the EU policy aiming to reduce their use (European directive 2009/128; Woo and Pepe, 2018). Among the different biocontrol options, the useof soil microorganisms to reduce crop losses and promote plant growth appears to be very promising. Indeed, many biological products (i.e., biopesticides, biostimulants, biofertilizers) already available on the market often contain beneficial fungi belonging to the genus *Trichoderma* (Woo et al., 2014; Woo and Pepe, 2018). Numerous strains of *Trichoderma* may have direct effects on plants, such as promotion of growth, nutrient uptake, efficiency of nitrogen use, seed germination rate and plant defenses against biotic and abiotic stress agents (Shoresh et al., 2010; Studholme et al., 2013; Lorito and Woo, 2015). In particular, as many other beneficial microbes (Pineda et al., 2015), some *Trichoderma* strains can activate Systemic Acquired Resistance (SAR) and/or Induced Systemic Resistance (ISR) (Segarra et al., 2007; Shoresh et al., 2010; Rubio et al., 2014; Martínez-Medina et al., 2017; Manganiello et al., 2018), whichconfer resistance against a wealth of phytopathogens (Van Wees et al., 2008). Indeed, *Trichoderma spp*. are widely used as biocontrol agents of plant pathogens (Lorito et al., 2010; Lorito and Woo, 2015; Manganiello et al., 2018), and are recognized as valuable Plant Growth Promoting Fungi (PGPFs) (Harman et al., 2004; Hermosa et al., 2012; Studholme et al., 2013; Mendoza-Mendoza et al., 2018). However, very few reports have addressed the role of these fungi in the modulation of plant defense responses against pest insects. Only in the last decade, the enhancement of indirect plant defense barriers against aphids was observed in plants colonized by *Trichoderma* (Guerrieri et al., 2004; Battaglia et al., 2013; Coppola et al., 2017a).

Plants have evolved both direct and indirect protection barriers to limit pest insects, such as the production of compounds able to directly interfere with physiology and reproduction of herbivores (i.e., direct defense), or to attract their natural enemies and exploit the ecological service they provide (i.e., indirect defense) (Walling, 2000; Kessler and Baldwin, 2002). The signals and the defense molecules locally produced at the damage site are often systemically circulated throughout the plant, while the released volatile blend modulates the interactions not only with higher trophic levels (i.e., herbivores and their natural enemies), but also with neighboring healthy plants, which can perceive the “alarm messages” emitted by injured conspecifics (Conrath, 2011; Coppola et al., 2017b).

The titer of different plant hormones, such as salicylic acid (SA), ethylene (ET), and jasmonic acid (JA), is modulated by damage and the induced changes activate hormone-dependent key-regulators of downstream plant defense pathways (Pieterse and van Loon, 1999, 2004; Thaler et al., 2002). Biotrophic pathogens generally trigger the SA pathway, while necrotrophic colonization activates both of ET and JA pathways (Pieterse and van Loon, 1999; Walling, 2000; Harman et al., 2004). Insect chewing on plant tissues mostly induce the JA pathway (Schilmiller et al., 2007; Pieterse et al., 2012), while sap feeders predominantly activate SA-dependent responses (Walling, 2000, 2008). However, these signaling pathways are tightly interconnected to allow a fine control of optimal resource allocation between plant growth and response to environmental stress agents; the underlying network of cross-modulating pathways is often manipulated by plant enemies to evade or actively suppress the defense barriers (Pieterse et al., 2012). The antagonism between SA, ET, and JA pathways, dictated by the need to prioritize the response against a specific type of biotic stressor, has been demonstrated in many plant species (Reymond and Farmer, 1998; Spoel and Dong, 2008; Pieterse et al., 2012). However, numerous attackers can exploit this antagonism to their own benefit by activating responses to which they are not sensitive, thus preventing/limiting energy investments in defense pathways detrimental for them (Erb et al., 2012).

This intricate network of molecular interactions among different stress agents has a further layer of complexity, which is added by the soil and plant-associated microbiota, deeply influencing the overall plant response (Berendsen et al., 2012; Bulgarelli et al., 2013; Pineda et al., 2017). Plants, along with the associated microbiota in the surrounding environment, are therefore the living milieu in which a complex network of multitrophic interactions among pests/and beneficial organisms takes place. Then, the molecular mechanisms driving plant defense responses against pests and in presence of a beneficial micro-organism can only be understood if analyzed at metaorganism level. These studies will shed light on the co-evolutionary forces shaping insect communities on plants and will offer valuable insights for developing novel strategies of pest control that can mimic and/or modulate plant defense responses.

Here we pursue this objective by investigating transcriptomic and metabolomic changes induced in *Solanum lycopersicum* (cv “Dwarf San Marzano”) by the beneficial fungus *Trichoderma harzianum* strain T22, and a pest insect, the aphid *Macrosiphum euphorbiae* (Thomas), when applied to the experimental plants alone or in combination.

## Materials and Methods

### Fungal Cultures and Insects

*T. harzianum* strain T22 (T22) was maintained on potato dextrose agar (PDA; Hi Media) slants at room temperature and regularly sub-cultured. Conidia were collected from the surface of sporulating fungal cultures (5–7 d) in sterile distilled water, and adjusted to a concentration of 10^7^ spores mL^−1^.

The aphid *M. euphorbiae* was reared on tomato “Dwarf San Marzano” (hereafter indicated as SM), in a climatic chamber at 20 ± 1°C, 65 ± 10% RH, photoperiod of 16:8 hr light/dark.

### Plant Material and Treatments

Seeds of *Solanum lycopersicum* cv “Dwarf San Marzano” (SM) were surface-sterilized in 2% (v/v) sodium hypochlorite for 20 min, then thoroughly rinsed in sterile distilled water. Seeds were treated with the fresh spore suspension of *T. harzianum* T22, as a seed coating (conc. 10^7^ spores mL^−1^), or with water as a control treatment (CTRL); stirred frequently to cover the seed surface uniformly, left to air dry for 24 h, then stored at 4°C until use. Treated seeds were germinated on wet sterile paper disks in the dark, in an environmental chamber at 24Â°C, then transplanted to sterile potting soil upon root emergence and grown in controlled conditions at 20 ± 2°C, with a photoperiod of 16:8 h light/dark. After 3 weeks, tomato seedlings were transplanted to 14-cm diameter plastic pots containing sterilized soil and grown for 2 weeks under the same environmental conditions. Plants from the T22 coated seeds also received a supplementary watering with the T22 spore suspension (20 mL; 10^7^sporemL^−1^) after the transplant and, after that, on a weekly basis. Leaf samples were collected from all tomato plants (T22 and CTRL) 2 weeks after the last T22 watering treatment.

### Aphid Infestation and Bioassay

A clonal population of *M. euphorbiae* was reared on SM in an environmental chamber at 20 ± 2°C, 65 ± 5% RH and a 16:8 h light/dark photoperiod. For the transcriptomic analysis, the control and *T. harzianum*-treated plants (T22) subjected to aphid infestation after 4 weeks of growth under the same environmental conditions indicated above. Three biological replicates, both for CTRL and T22 plants, were caged and infested with synchronized 1-day-old nymphs of *M. euphorbiae*. Five aphids per plant were settled and allowed to feed for 48 h, then removed from the plant in order to collect aphid-free leaf samples for the subsequent RNA extraction (samples named as “Aph” or “T22Aph”).

For the aphid longevity assay, 10 plants for each CTRL or T22 treatment were infested with 5 newly born first instar nymphs of *M. euphorbiae*. The presence of aphids and of shed exuviae, as an indicator of molting occurrence, was daily monitored. Survival curves were compared by LogRank analysis.

### RNA-Seq

Fully expanded leaves (5 leaves) of 3 tomato plants for each treatment were used for total RNA extraction: leaf samples treated with T22 (T22), infested by aphids (Aph), treated with T22 and infested by aphids (T22Aph), and untreated (CTRL). Total RNA was extracted using the Plant RNeasy mini kit (Qiagen) according to manufacturer's protocol. Samples were analyzed with the 2,100 Bioanalyzer system (Agilent Technologies) for size, quantification, and quality control of RNA. Only samples with a 260/280 nm absorbance >1.8 and a 260/230 nm absorbance >2 were sequenced. Three biological replicates were used for each experimental condition and controls. Total RNA (8 μg) of each sample was used for the library preparation and sequencing by an external sequencing service. A paired-end sequencing (2 × 30 Million of reads) on Illumina HiSeq 2,500 platform was chosen. RNA-Seq raw sequences were cleaned using Trim Galore package [http://www.bioinformatics.babraham.ac.uk/projects/trim_galore/]. Low-quality bases were trimmed from the sequences and the adapter sequences were removed by Cutadapt (Martin, 2011); default parameters for the pair-end sequences were used. Finally, if one of the pairs was filtered out due to the cleaning procedure, the other pair was also discarded from the downstream analyses.

The cleaned sequences were then mapped on the tomato genome (version 2.50) using Bowtie version 2.1.0 (Langmead and Salzberg, 2012) and Tophat version 2.0.8 (Kim et al., 2013). Quantification of the reads abundance per each gene (exon level) available from iTAG gene annotation (version 2.5) was done using AIR (https://transcriptomics.sequentiabiotech.com/).

To identify the set of Differentially Expressed Genes (DEGs) between the diverse experimental conditions, two different statistical approaches were used: the Negative Binomial test implemented in DESeq package (Anders and Huber, 2010) and the Negative Binomial test and Generalized Linear Model (GLM), as implemented in EdgeR package (Robinson et al., 2010), were used considering false discovery rate (FDR) < = 0.05. The data from the two methods were compared and where the values intersected, these results were considered and selected to compile the datasets used for the analysis of the differentially expressed genes.

RNA-Seq validation was carried out by Real Time RT-PCR, measuring the transcript levels of selected DEGs. Gene expression analysis was carried out using 2 technical replicates for each of the 3 biological replicates per sample. Relative quantification of gene expression was carried out using the 2^−ΔΔ*Ct*^ method (Livak and Schmittgen, 2001). The housekeeping gene EF-1α was used as endogenous reference gene for the normalization of the expression level of the target genes (Marum et al., 2012; Müller et al., 2015). Ten couples of primers were used to analyze each treatment condition. Primers and their main features are reported in the Supplementary Table 1.

### Functional Annotation

GO and GOslim annotations were downloaded from the Biomart section of Ensembl Plant version SL2.50 (2014-10-EnsemblPlants) (Kinsella et al., 2011). Moreover, GO was used for GO enrichment of all DEGs together and DEGs UP or DOWN regulated, independently. The analysis was carried out by the Goseq Bioconductor package (Young et al., 2010) (method “BH,” FDR ≤ 0.05).

Mapping of some enzymatic activities into specific molecular pathways was acquired from the KEGG database.

### Semi-polar Metabolome Analysis

LC-ESI(+)-MS analysis of the leaf primary and secondary semi-polar metabolome was performed as previously described (Alboresi et al., 2016; Fasano et al., 2016) with slight modifications: 5 mg of freeze-dried, homogenized leaf powder were extracted with 0.75 mL cold 75% (v/v) methanol, 0.1% (v/v) formic acid, spiked with 10 μg/ml formononetin. After shaking for 40'at 20 Hz using a Mixer Mill 300 (Qiagen), samples were centrifuged for 15' at 20,000 g at 4°C; 0.6 mL of supernatant were removed and transferred to HPLC tubes. For each genotype, 4 independent biological replicates, consisting of 4 plants each, were analyzed; for each biological replicate, at least one technical replicate was carried out. LC-MS analyses were carried out using an LTQ-Orbitrap Discovery mass spectrometry system (Thermo Fisher Scientific) operating in positive electrospray ionization (ESI), coupled to an Accela U-HPLC system (Thermo Fisher Scientific, Waltham, MA). Liquid chromatography was carried out using a Phenomenex C18 Luna column (150 × 2.0 mm, 3 μm) and the mobile phase was composed by water −0.1% formic acid (A) and acetonitrile −0.1% formic acid (B). The gradient was: 95%A:5%B (1 min), a linear gradient to 25%A:75%B over 40 min, 2 min isocratic, before going back to the initial LC conditions in 18 min. Five microliter of each sample were injected and a flow of 0.2 mL was used throughout the LC runs. Detection was carried out continuously from 230 to 800 nm with an online Accela Surveyor photodiode array detector (PDA, Thermo Fisher Scientific, Waltham, MA). All solvents used were LC-MS grade quality (CHROMASOLV® from Sigma-Aldrich). Metabolites were quantified in a relative way by normalization on the internal standard (formononetin) amounts. ESI-MS ionization was performed using the following parameters: capillary voltage and temperature were set at 20V and 280°C; sheath and aux gas flow rate at, respectively, 30 and 20. Spray voltage was set to 3.5 kV and tube lens at 80 V. Targeted metabolite identification was performed by comparing chromatographic and spectral properties with authentic standards and reference spectra, in house database, literature data, and on the basis of the m/z accurate masses, as reported in the Pubchem database (http://pubchem.ncbi.nlm.nih.gov/) for monoisotopic mass identification, or on the Metabolomics Fiehn Lab Mass Spectrometry Adduct Calculator (http://fiehnlab.ucdavis.edu/staff/kind/Metabolomics/MS-Adduct-Calculator/) in the case of adduction detection.

Untargeted metabolomics was performed using the SIEVE software (Thermofisher scientific). After chromatogram alignment and retrieve of the all the detected frames (e.g., ions), an ANOVA + *t*-test statistical analysis was carried out to identify differentially accumulated molecules. Finally, a series of public metabolomic databases (KEGG, HMD, Golm Metabolome Database, PlantCyc) were interrogated and a list of tentative IDs was obtained. Further validation steps included isotopic pattern ratio (IPR), mass fragmentation when available and literature search.

Principal component analysis (PCA) of untargeted semipolar metabolome was performed by using the SIEVE software (Thermofisher Scientific). Venn diagram representation of differentially accumulated metabolites (DAMs) was performed using the Venny 2.1 software [Oliveros, J. C. (2007–2015)]. Metabolite heat maps and hierarchical clustering were build and colored by using the GENE-E software (http://www.broadinstitute.org/cancer/software/GENE-E/) and as previously described (Diretto et al., 2010).

## Results

### *Trichoderma harzianum* T22 Promotes Tomato Defense Against *M. euphorbiae*

**Figure 1:**
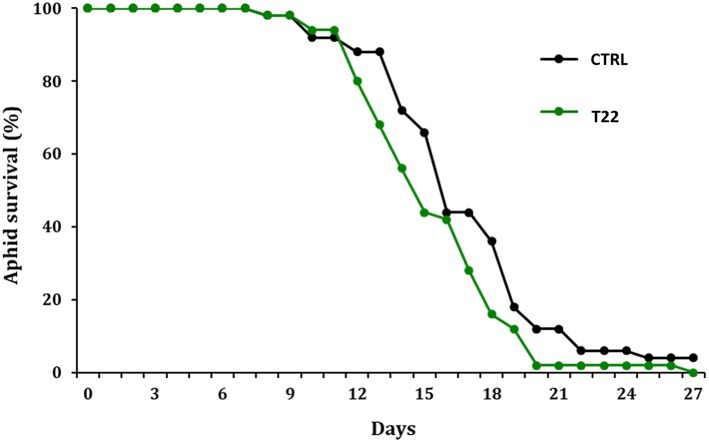
Effect of *T. harzianum* T22 on aphid survival over time. Survival curves (percentage) of *M. euphorbiae* reared on the untreated water control and the *T. harzianum* T22 treated tomato plants are significantly different, *p* < 0.05 (LogRank test).

The treatment of tomato plants with *T. harzianum* T22 negatively influenced the survival rate of *M. euphorbiae*. T22 plants showed an increased level of resistance to aphid infestation, as indicated by the significant difference registered between the T22 survival curve and that of CTRL (LogRank analysis, χ^2^ = 4.72, *p* = 0.030, *df* = 1) (Figure 1).

### Plant Transcriptome Reprogramming Induced by *Trichoderma harzianum* T22 Root Colonization

In order to unravel the molecular mechanisms underlying the plant response to the combined action of *Trichoderma* infection and aphid infestation, transcriptomic and metabolomic analyses ofthe tomato cultivar “Dwarf San Marzano” were conducted by comparing *Trichoderma* treated and untreated plants, with and without aphid infestation.

Table 1 provides a general summary of differentially expressed genes for each treatment.

**Table 1:** General overview of the transcriptomic rearrangement of Dwarf San Marzano tomato plants imposed by experimental treatments compared to untreated SM plants.

	**T22**	**Aph**	**T22Aph**
**Total DEGs**	978	1804	1527
**Up-regulated**	515	625	602
**Down-regulated**	463	1179	925

*T22: treatment with T. harzianum T22, Aph: aphid infestation, T22 Aph: treatment with T. harzianum T22 followed by aphid infestation*.

**Figure 2:**
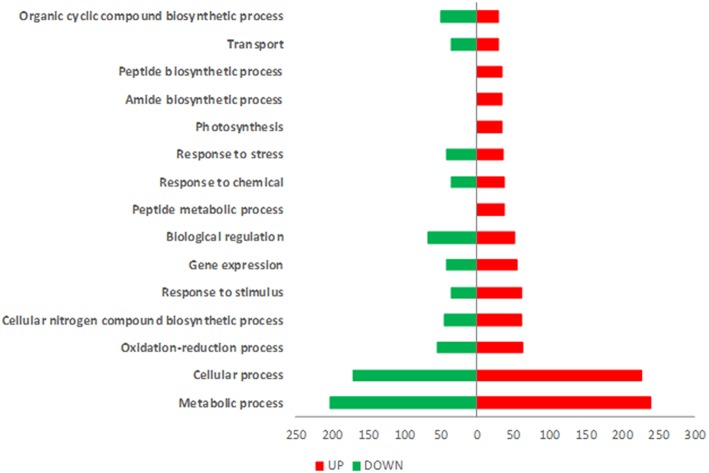
GOs distribution of differentially expressed genes in SM plants treated with *T.harzianum*T22. Gene Ontology (GO) terms associated with up-regulated (red bars) and down-regulated (green bars) genes based on the “Biological Process” ontological domain (sequence cut-off: 5%).

T22 plants showed a total of 978 differentially expressed genes (DEGs) of which 515 were up-regulated and 463 were down-regulated (Supplementary Tables 2A,B). The principal defense-related categories that may be linked to the induction in T22 treated plants of a precursor state of defense against insect attack are represented by “response to stress,” “transport” and “response to stimulus” (Figure 2). The most abundant enriched Gene Ontology (GO) terms in the ontological category “Biological Process” were associated with metabolic processes, photosynthesis-related mechanisms, oxidation-reduction processes and response to stress (Supplementary Figure 1).

Several genes included in these categories were up-regulated. A short list of these genes is reported in Table 2. Among them, the induction of genes associated with photosynthesis, chlorophyll biosynthesis and sequestration and biosynthetic processes, may be linked with T22 beneficial effects on tomato plant physiology The up-regulation of plant genes involved in early signals of defense responses against environmental cues as, for example, Serine/threonine-protein kinase, Leucine-rich repeat protein kinase, LRKs, Glutathione S-transferase, and others (listed in Supplementary Table 2A) was also observed. Similarly, the up-regulation of genes coding for transcription factors (TF), such as Ethylene responsive transcription factors (ERF), WRKY, MYB, and bZIP TF (Supplementary Table 2A), was registered. These genes are likely involved in plant defense priming (Conrath et al., 2015).

**Table 2:** Examples of tomato genes affected by Trichoderma T22 treatment.

**Gene ID**	**logFC**	**Gene description**
**PHOTOSYNTHESIS**		
Solyc03g114930.3	1,525559	Photosystem II reaction center PsbP family protein
Solyc09g064500.3	1,498658	Photosystem II reaction center Psb28 protei
Solyc06g060340.3	1,400894	Photosystem II subunit S
Solyc07g066150.1	1,153408	Photosystem I reaction center subunit V family protein
Solyc06g084045.1	1,119141	Photosystem II reaction center W
Solyc06g065490.3	1,117796	Photosystem II reaction center PsbP family protein
Solyc02g069450.3	1,097231	Photosystem I reaction center subunit III
Solyc08g006930.3	1,087737	Photosystem I reaction center subunit psaK
Solyc12g044280.2	1,064617	Photosystem I reaction center subunit VI
**BIOSYNTHETIC PROCESSES**		
Solyc01g056780.3	1,463119	50S ribosomal protein L34
Solyc11g066410.2	1,425305	50S ribosomal protein L9
Solyc02g068090.3	1,401681	30S ribosomal protein S21
Solyc06g082750.3	1,359268	50S ribosomal protein L17
Solyc11g068820.2	1,283545	50S ribosomal protein L27
Solyc04g079790.3	1,180683	30S ribosomal protein S9
Solyc04g074900.3	1,164445	40S ribosomal protein S21
Solyc07g062870.3	1,135344	30S ribosomal protein S20
Solyc09g097910.3	1,111600	30S ribosomal protein S1
**CHLOROPHYLL BIOSYNTHESIS AND SEQUESTRATION**		
Solyc08g062290.3	1,676942	Light-independent protochlorophyllide reductase subunit B
Solyc10g007320.3	1,376821	Uroporphyrinogen decarboxylase
Solyc10g077040.2	1,102132	Magnesium-protoporphyrin monomethyl ester
**PHENYLPROPANOID OR FLAVONOID SYNTHESIS**		
Solyc06g074710.1	−2,23399	Hydroxycinnamoyl-CoA shikimate/quinate hydroxycinnamoyl transferase
Solyc08g061480.3	−1,07068	Chalcone–flavonone isomerase
Solyc08g005120.3	−1,63331	Cinnamoyl-CoA reductase-like protein

*Values of Log2 Fold Change and gene description are indicated*.

**Figure 3:**
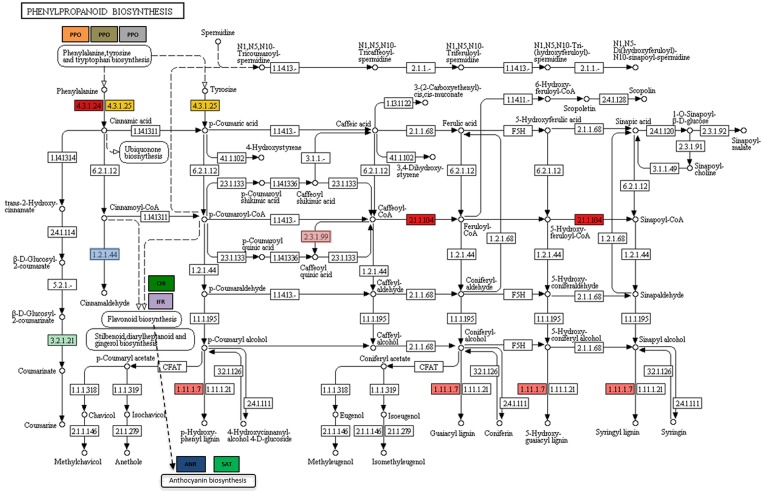
Schematic diagram of the phenylpropanoid biosynthesis pathway as determined by DEGs in plants treated with T22 and queried to a KEGG database. The enzymes evidenced in color are encoded by genes found in the up-regulated DEGs of the tomato. A correspondence between enzymes and DEGs is shown in Table 2.

As expected, the up-regulation of markers of SA (i.e., chitinase and1,3-B glucanase) and JA pathways (i e., metallocarboxypeptidase inhibitor and, Type I serine protease inhibitor; Supplementary Table 2A) were also observed. Overall, these data show that *Trichoderma* colonization of tomato plants positively affects several metabolic pathways, consistently with previous observations in tomato and other plant species (Alexandru et al., 2013; Mohapatra and Mittra, 2016; Ban et al., 2018). Since phenylpropanoids contribute to plant defenses (both direct and indirect) to insect herbivores, genes encoding for key-enzymes associated with phenylpropanoids biosynthesis were searched by using DEGs in a query to a KEGG database; in addition, a manual curation to enrich the aforementioned list was performed. Transcripts coding for 10 enzymes were retrieved among the DEGs that were up-regulated and the correspondence found between the enzymes and the gene identifiers is listed in Table 3. As shown in Figure 3, the enzymes act in different stages of phenylpropanoid biosynthesis, catalyzing key-steps as phenylalanine conversion in cinnamic acid (*Phenylalanine ammonia-lyase (PAL)* and *Caffeoyl-CoA O-methyltransferase*), or catalyzing final branches for lignin production (i.e., *Peroxiredoxin*) and anthocyanin synthesis and modification (*Anthocyanin 5-aromatic acyl transferase* and *Anthocyanidin reductase*). Interestingly, a transcript coding for *PAL* is greatly up-regulated in T22 samples. In addition, a series of genes coding for enzymes involved in early phenylpropanoid or flavonoid synthesis resulted down-regulated (Table 2). Overall, these data show that T22 colonization strongly affects and remodels phenylpropanoid pathway.

**Table 3:** Differentially expressed genes involved in phenylpropanoid biosynthesis.

**Enzyme ID**	**Gene ID**	**logFC**	**Gene description**
4.3.1.24	Solyc03g036470.2	4,802883	Phenylalanine ammonia-lyase
4.3.1.25			
2.1.1.104	Solyc02g093250.3	1,099098	Caffeoyl-CoA O-methyltransferase
1.11.1.7	Solyc06g082420.3	1,179331	Peroxidase
		1,476077	Peroxiredoxin
3.2.1.21	Solyc01g060020	1,892951	β-1,3-glucanase
	Solyc02g086700	1,878683	
Manual curation	Solyc10g008680.2	2,199016	Anthocyanin 5-aromatic acyltransferase (5AT)
Manual curation	Solyc10g009507.1	1,533819	Anthocyanidin reductase (ANR)
Manual curation	Solyc01g067290.2	1,270974	Isoflavone reductase-related family protein (IFR)
Manual curation	Solyc08g074620.3	1,113982	polyphenoloxidase precursor (PPO)
Manual curation_2.3.1.99	Solyc06g074710.1	−2,234000	hydroxycinnamoyl-CoAshikimate/quinate hydroxycinnamoyl transferase
Manual curation_2.1.1.104	Solyc03g032220.3	−1,286107	Caffeoyl-CoA O-methyltransferase
Manual curation	Solyc08g074682.1	−1,236095	polyphenoloxidase precursor (PPO)
Manual curation_ 1.2.1.44	Solyc08g005120.3	−1,633312	Cinnamoyl-CoA reductase-like protein
Manual curation	Solyc08g061480.3	−1,070684	Chalcone—flavonone isomerase (CHI)
Manual curation	Solyc08g074683.1	−1,310960	polyphenoloxidase precursor (PPO)

*Enzyme and gene identifiers, fold change and gene description are listed. Colors associated to enzymes refers to Figure 3*.

### Plant Transcriptome Reprogramming Induced by Aphids

Studies on tomato-*M. euphorbiae* interaction and on the relative transcriptomic changes have been already reported, although using other tomato cultivars, different time points and diverse transcriptomic approaches (Avila et al., 2012; Coppola et al., 2013). Here we carried out a transcriptomic study of the SM cultivar challenged with *M. euphorbiae* for 48 h, through RNA-Seq approach.

**Figure 4:**
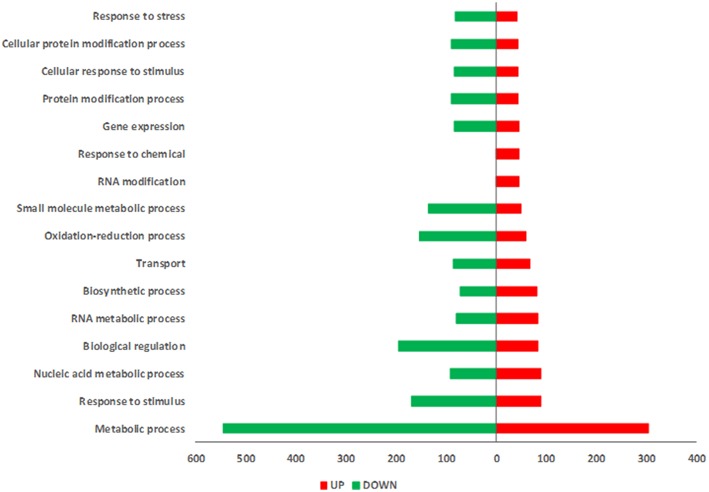
GOs distribution of differentially expressed genes in SM plants infested by*M. euphorbiae*. Gene Ontology (GO) terms associated with up-regulated (red bars) and down-regulated (green bars) genes based on the “Biological Process” ontological domain (sequence cut-off: 5%).

Tomato plants infested by *M. euphorbiae* showed 625 up-regulated and 1179 down-regulated transcripts (Supplementary Table 3). Major GO categories associated with plant defense were “response to stress,” “response to stimulus” and “oxidation-reduction process” (Figure 4). The distribution and the enrichment analysis of GO terms associated with DEGs induced by aphid infestation underlined the predominance of categories related to the regulation of gene expression as “RNA methylation,” “ncRNA processing,” “Ribosome assembly,” “rRNA metabolic process,” “translation,” “mRNA cleavage,” “defense response to bacterium” (Supplementary Figure 2). The increase of several transcripts coding for kinase/phosphatase/receptor-like kinase as well as of transcripts coding for proteins involved in oxidative burst and scavenging was observed (Supplementary Table 3A). Genes coding for several classes of pathogenesis-related proteins (PR) (PR5, PR10, Chitinase, Subtilisin), genes associated with salicylic acid and genes involved in ethylene signaling were also up-regulated upon aphid attack (Supplementary Table 3A). A large number of DEGs were down-regulated (Supplementary Table 3B and Table 4), including key genes of plant immunity, such as MAP Kinases and WRKY. Interestingly, a strong down-regulation was observed for transcripts associated with JA pathway, as those coding for lipoxygenases and protease inhibitors. Other down-regulated transcripts code for glycosyltransferases and genes associated with terpene production, such as sesquiterpene synthase 1 and geranylgeranyl reductase.

**Table 4:** Example of defense-related down-regulated genes by aphid infestation.

**Gene ID**	**logFC**	**Gene description**
**ETHYLENEBIOSYNTHESIS AND SIGNALING**		
Solyc08g078180.1	−2,71739	Ethylene Response Factor A.1
Solyc04g071770.3	−1,81979	Ethylene-responsive transcription factor
**JA SIGNALING PATHWAY**		
Solyc03g020040.3	−2,64679	Pin-II type proteinase inhibitor 69
Solyc01g099160.3	−2,52785	Lipoxygenase
Solyc08g014000.3	−2,09537	Lipoxygenase A
Solyc11g022590.1	−1,94405	Trypsin inhibitor-like protein precursor
Solyc00g187050.3	−1,89562	Leucine aminopeptidase 2
Solyc08g074682.1	−1,87224	Polyphenoloxidase precursor
Solyc07g007250.3	−1,8422	Metallocarboxypeptidase inhibitor
Solyc09g084450.3	−1,79009	Proteinasi inhibitor I
Solyc12g010030.2	−1,77364	Leucine aminopeptidase
Solyc01g006540.3	−1,42694	Lipoxygenase C
Solyc09g008670.3	−1,38363	Threonine deaminase
Solyc04g077650.3	−1,27008	Serine carboxypeptidase
Solyc03g118540.3	−1,25772	Jasmonate ZIM-domain protein 7b
**ISOPRENOID AND TERPENOID PATHWAY**		
Solyc06g059930.3	−2,17858	Sesquiterpene synthase 1
Solyc10g005410.3	−2,1206	Terpene synthase
Solyc08g005710.3	−1,57829	Terpene synthase 41
**PHENYLPROPANOID PATHWAY**		
Solyc06g074710.1	−3,68386	Hydroxycinnamoyl-CoAshikimate/quinate hydroxycinnamoyltransferase
Solyc06g084050.3	−2,39253	Isochorismatesynthase 2
**POLYAMINE BIOSYNTHESIS**		
Solyc10g009380.3	−1,03958	Arginine N-methyltransferase
Solyc03g098300.1	−2,12424	Ornithine decarboxylase 2
Solyc01g010050.3	−1,77437	S-adenosylmethionine decarboxylase proenzyme
Solyc07g039310.1	−1,42553	Polyamineoxidase 5
**SUGAR METABOLISM**		
Solyc07g065900.3	−1,71894	Fructose-bisphosphate aldolase
Solyc09g092130.3	−1,0955	Sucrose-phosphate synthase
Solyc02g071590.2	−1,26851	Trehalose-6-phosphate synthase
Solyc03g112500.3	−7,11877	Raffinose synthase
**AMINO ACID PATHWAY**		
Solyc07g054280.1	−3,48542	Tyrosine decarboxylase
Solyc09g008670.3	−1,38363	Threonine deaminase
Solyc10g005320.3	−1,36504	Tryptophan synthase
Solyc06g019170.3	−2,25121	Delta-1-pyrroline-5-carboxylate synthetase
**CHLOROPHYLL METABOLISM AND PHOTOSYNTHESIS-RELATED GENES**		
Solyc01g060085.1	−3,38289	Ribulose bisphosphate carboxylase large chain
Solyc07g062530.3	−2,86058	Phosphoenolpyruvate carboxylase 2
Solyc12g013710.2	−2,81926	Light dependent NADH:protochlorophyllide oxidoreductase 1
Solyc03g005790.2	−2,65938	Chlorophyll a-b binding protein
Solyc04g006970.3	−2,1064	Phosphoenolpyruvate carboxylase
Solyc06g053620.3	−1,91070	Phosphoenolpyruvate carboxylasekinase 2
Solyc09g011080.3	−1,53137	Ribulose bisphosphate carboxylase/oxygenase activase
Solyc02g086650.3	−1,30017	Phosphoenolpyruvate/phosphate translocator
Solyc10g077040.2	−1,07567	Magnesium-protoporphyrin monomethyl ester cyclase

*Values of Log2 Fold Change and gene description are indicated*.

Overall, at primary metabolism level, aphid infestation strongly repressed transcripts of enzymes associated with sugar (i.e., *Fructose-bisphosphatealdolase* and *Sucrose synthase*) and amino acid (i.e., *Threonine deaminase* and *Tryptophan synthase*) pathways, which are involved in the plant defense responses against biotic and abiotic stresses (Conklin and Last, 1995; Brader et al., 2001; Wilkinson et al., 2001; Chen et al., 2005; Tauzin and Giardina, 2014; Lv et al., 2017). Several other down-regulated genes were involved in photosynthetic activities, chlorophyll biosynthesis, polyamine and phenylpropanoids-related metabolism (Table 4).

### Plant Transcriptome Reprogramming Induced by *Trichoderma harzianum* T22 Root Colonization and Aphid Infestation

**Figure 5:**
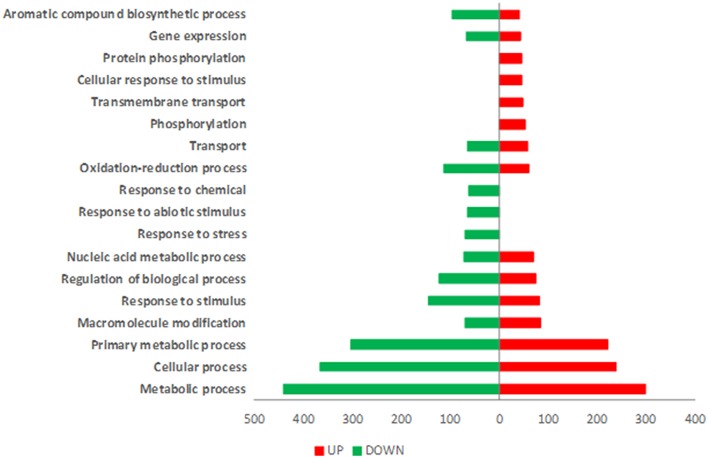
GOs distribution of DEGs in T22-Aph plants, first treated with *T. harzianum* T22 and subsequently infested with aphids (SMT22 Aph). Gene Ontology (GO) terms associated with up-regulated (red bars) and down-regulated (green bars) genes based on the “Biological Process” ontological domain (sequence cut-off: 5%).

In order to assess the impact of *T. harzianum* T22 on tomato defense response against aphids, the transcriptome of tomato plants treated with *Trichoderma* and subsequently infested by aphids (T22Aph) was analyzed. T22Aph transcriptome reprogramming involved 1527 transcripts: 602 up- and 925 down-regulated (Supplementary Table 4). *Trichoderma* colonization strongly affected GO categories involved in plant metabolism and stress response during aphid infestation (Figure 5). The enrichment analysis was performed in order to underline significant over-represented GO categories relative to Biological Process ontological domain. Interestingly, some enriched GO term categories were associated with direct and indirect defenses, as they include genes involved in isoprenoid biosynthesis, induced systemic resistance and JA-mediated signaling pathway (Supplementary Figure 3; Supplementary Tables 4A,B). An example of defense-related DEGs is shown in Table 5. Among early signals, calmodulin-binding proteins and Ca^2+^ transporters were also over-represented as several classes of kinases and receptor-kinases (serine/threonine kinases, receptor-like kinases, LRR-RLKs, MAPKKK). Furthermore, transcripts related to ROS production and scavenging such as GST, peroxidases, oxidoreductases, catalase, superoxide dismutase, and detoxification protein were up-regulated. Other up-regulated genes coded for Lipoxygenases, involved in early stages of JA biosynthesis, Polyphenol oxidase (PPO), Leucine aminopeptidase (LapA), and proteinase inhibitor (MCPI) involved in later stages of defense besides several classes of defense genes-related TF (GRAS, WRKY, MYB, bZIP). Moreover, a transcript encoding for a cysteine protease inhibitor (Multicystatine), associated with aphid growth inhibition (Rahbé et al., 2003; Emani, 2018), was up-regulated while the number of down-regulated transcripts encoding proteinase inhibitors was reduced in comparison with what observed following aphid infestation.

**Table 5:** Group of defense-related DEGs identified in San Marzano plants treated with Trichoderma T22 and infested by aphid.

**Gene ID**	**logFC**	**Gene description**
**ISOPRENOID PATHWAY**		
Solyc07g052135.1	5,569388	Sesquiterpenesynthase
Solyc11g011240.1	1,544086	geranylgeranylpyrophosphatesynthase 1
**ETHYLENEBIOSYNTHESIS AND SIGNALING**		
Solyc05g051180.2	7,829771	Ethylene-responsive transcription factor
Solyc11g045520.2	2,490749	1-aminocyclopropane-1-carboxylate oxidase-like protein
Solyc05g051200.1	2,049015	Ethylene-responsive factor 1
Solyc08g008305.1	1,688514	Ethylene-responsive transcription factor ERF061
**SALICYLIC ACID BIOSYNTHESIS AND SIGNALING**		
Solyc08g080670.1	2,301690	Pathogenesis-related 5-like protein
Solyc08g080660.1	2,286811	Osmotin-like protein
Solyc07g009500.2	2,224245	Chitinase
Solyc09g090990.2	2,162828	PR10 protein
Solyc01g087840.3	2,161848	Subtilisin-like protease
Solyc08g079900.3	1,597817	subtilisin-like protease
Solyc01g005230.3	1,590776	S-adenosyl-L-methionine-dependent methyltransferase superfamily protein
**JA SIGNALING PATHWAY**		
Solyc08g029000.3	3,645744	Lipoxygenase
Solyc00g071180.3	2,670684	Multicystatin
Solyc06g061230.3	1,805861	Metallocarboxypeptidaseinhibitor
Solyc01g091170.3	1,389207	arginase 2 ARG2
Solyc12g010030.2	1,295502	Leucine aminopeptidase
Solyc08g074620.3	1,144778	polyphenoloxidase precursor
Solyc06g048820.1	1,020496	Wound-inducedprotein 1
**PHENYLPROPANOID PATHWAY**		
Solyc03g036470.2	−6,60171	Phenylalanine ammonia-lyase
Solyc09g091510.3	−2,13284	chalconesynthase 1
Solyc05g053550.3	−1,92049	chalconesynthase 2
Solyc11g013110.2	−1,49002	Flavonolsynthase
Solyc02g085020.3	−1,21211	dihydroflavonol 4-reductase

*Values of Log2 Fold Change and gene description are indicated*.

A strong impact on hormone-controlled defense pathways was observed: ethylene biosynthesis and signaling as well as salicylic acid biosynthesis and signaling were up-regulated in T22Aph plants (Supplementary Table 4; Table 5). Notably, compared to T22, T22Aph were characterized by a strong down-regulation of key-steps in the phenylpropanoid pathway (Table 5).

For a selected number of genes, transcript quantification was confirmed by Real Time RT-PCR (Supplementary Figure 4).

#### Key Genes Regulated by the Interaction T22-Tomato-Aphid

**Figure 6:**
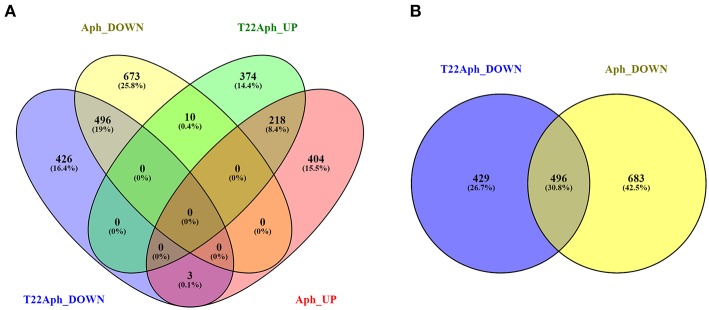
Venn diagram visualization of DEGs. **(A)** DEGs of tomato plants infested by the aphid *M. euphorbiae* (Aph) are crossed to DEGs of tomato plants inoculated with *T. harzianum* T22 and subsequently infested by aphids (T22Aph). **(B)** Focus on the intersection of down-regulated genes of Aph and T22Aph plants.

In order to assess the contribution of *T. harzianum* T22 in the priming of defenses against aphids, genes specifically regulated in the tripartite interaction were analyzed. Supplementary Tables 5A,B list unique genes modulated in their expression during the T22-Tomato-Aphid interaction (T22Aph samples, Figure 6A). These genes are specifically induced by aphid challenge in presence of *Trichoderma* priming. Among the up-regulated ones, genes involved in ethylene biosynthesis and signaling emerge (Table 6), as well as those associated with amino acid metabolism (*asparagine synthase 1, glutamate receptor 1.2, proline dehydrogenase*). Unique down-regulated genes (Supplementary Table 5B; Table 6) included several members of WRKY family of transcription factors, known for their promotion of JA signaling in the negative interplay with SA pathway (Li et al., 2004; Takatsuji, 2014).

**Table 6:** Example of DEGs modulated by aphid challenge in presence of Trichoderma priming.

**Gene ID**	**logFC**	**Gene description**
**ETHYLENE BIOSYNTHESIS AND SIGNALING**		
Solyc12g056590.2	3,27892	Ethylene Response Factor D.2
Solyc06g065820.3	2,651662	Ethylene Response Factor H.1
Solyc11g045520.2	2,490749	1-aminocyclopropane-1-carboxylate oxidase-like protein
Solyc03g111620.1	2,051024	S-adenosyl-L-methionine-dependent methyltransferase superfamily protein
Solyc05g052030.1	1,803781	Ethylene responsive factor 4
Solyc08g008305.1	1,688514	Ethylene-responsive transcription factor ERF061
Solyc08g014120.3	1,60402	Ethylene responsive protein 33
**WRKYS FAMILY OF TF**		
Solyc09g010960.3	−3,58083	WRKY transcription factor 49
Solyc08g067360.3	−2,65237	WRKY transcription factor 45
Solyc03g007380.2	−2,44306	WRKY transcription factor 52
Solyc04g051690.3	−2,19968	WRKY transcription factor 51
Solyc08g067340.3	−1,89527	WRKY transcription factor 46
Solyc08g062490.3	−1,22158	WRKY transcription factor 50
Solyc08g082110.3	−1,16835	WRKY transcription factor 54
Solyc09g015770.3	−1,07513	WRKY transcription factor 81
Solyc09g014990.3	−1,07294	WRKY transcription factor 33

*Values of Log2 Fold Change and gene description are indicated*.

Figure 6A shows that aphid repression of tomato genes (1179 down-regulated genes in Aph) was reduced by *T. harzianum* T22 colonization in T22Aph (925 down-regulated genes in T22Aph).

The intersection between down-regulated genes of Aph and T22Aph samples is shown through a Venn diagram representation (Figure 6B). Common genes repressed in both conditions are listed in Supplementary Table 5C. A large group of genes of phenylpropanoid pathways (i.e., *phenylalanine ammonia-lyase, caffeoyl-CoA O-methyltransferase*, and others) resulted down-regulated in both conditions. Interestingly, a large number of glycosyltransferases resulted strongly repressed in both conditions, indicating that they could represent a peculiar aspect of tomato-aphid interaction, independently from *T. harzianum* T22 influence (Supplementary Table 5C). Genes specifically repressed in the bipartite interaction (Aph) are 683 (Figure 6B; Supplementary Table 5D) and include JA-related genes as those coding for Phospholipase, Lipoxygenases A, C and D, Leucine aminopeptidase A1 and several classes of proteinase inhibitors (Table 5). In order to assess if *T. harzianum* T22 is able to overturn the expression of aphid-repressed genes in tomato, the 683 specifically down-regulated transcripts in Aph samples were compared with genes induced by T22 (T22; Supplementary Table 2A), underlining an overturning of the expression of three genes listed in Table 7. The transcription factor bHLH may be associated with JA signaling (Zhou and Memelink, 2016) while the steroid dehydrogenase, involved in steroid and squalene biosynthesis, is a precursor of triterpenes. Finally, the GDSL esterase/lipase belong to a very large subfamily of lipolytic enzymes.

**Table 7:** Overturned expression of genes in dipartite interactions (tomato-aphid and tomato-T22).

**Gene ID**	**T22 (Log2FC)**	**Aph (Log2FC)**	**Gene description**
Solyc03g118310.3	1,02474507	−1,95569	bHLH transcription factor 083
Solyc11g006300.2	1,42671042	−1,12261	3-oxo-5-alpha-steroid 4-dehydrogenase family protein
Solyc01g099030.3	1,06934649	−1,11098	GDSL esterase/lipase

*Gene ID, Log2 Fold change in T22 and Aph samples, respectively, and gene description are listed*.

### Metabolomic Analysis

In order to analyse the downstream effects of the transcriptomic reprogramming induced by *M. euphorbiae* attack, in the absence or presence of the antagonist fungus *T. harzianum* T22, we performed a global metabolic profiling of the leaf semi-polar fraction by LC-ESI(+)-MS (for more details, see “Materials and methods). First of all, to gain a general overview of the metabolic changes occurring under the different experimental conditions, we carried an untargeted metabolomics analysis, using the SIEVE software (Thermo Fisher Scientific). Through alignment of all mass chromatograms with the subsequent retrieval of all detected ions, we built a 3D Principal Component Analysis (PCA) diagram (Supplementary Figure 5), which showed a clear separation of the leaves treated with *T. harzianum* and, to a lower extent, infested by aphids in the presence of the fungus. To investigate the changes of known tomato leaf metabolites, we then performed a targeted metabolomic analysis in which we quantified, in a relative way, 135 metabolites involved in primary (amino acids, amines, sugars, organic acids, lipids, vitamins, etc) and secondary (alkaloids, amides, phenylpropanoids, isoprenoids) pathways. The complete metabolite dataset is reported in Supplementary Table 7 and Supplementary Figure 6, while the lists of the differentially accumulated metabolites (DAM) in each comparison (T22/CTRL, Aph/CTRL, and T22Aph/T22) are reported in Supplementary Table 6.

**Figure 7:**
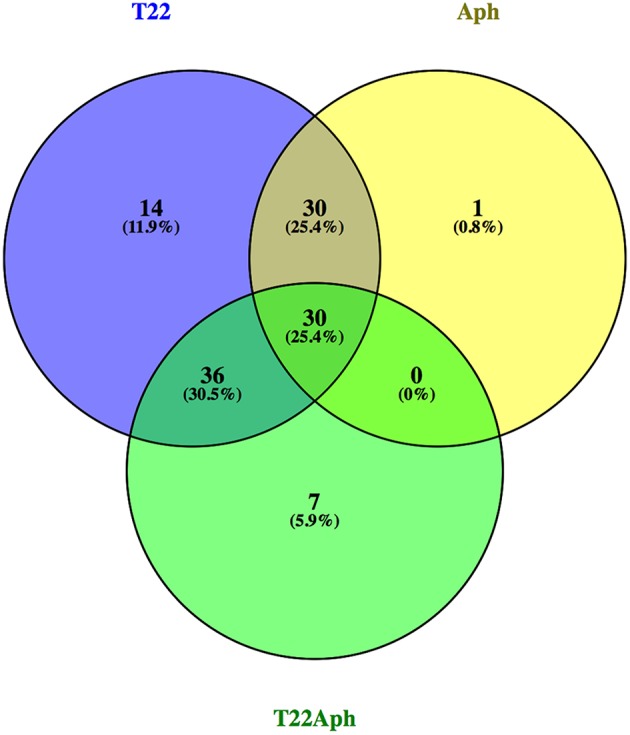
Venn diagram visualization of differentially accumulated metabolites in three comparisons between tomato plants: infested with aphids vs. water control (Aph vs. CTRL); treated with T22 vs. water control (T22 vs. CTRL); treated with T22 plus infested with aphid vs. treated with T22 (T22 Aph vs. T22).

Heatmap visualization was used as first attempt to understand the real impact of the aphid and fungus treatments on the leaf metabolome (Supplementary Figure 6). Globally, most of the alterations in leaves grown in the presence of *M. euphorbiae* or *T. harzianum* were of negative sign (e.g., lower levels in the treated over the control) and particularly affected secondary metabolism (alkaloids and phenylpropanoids). We used Venn diagram visualization (Figure 7; Supplementary Table 8) to highlight the number of common and specific DAMs in relation to the three interactions under study: interestingly, 29 metabolites resulted present in all the comparisons and 17 out of them displayed variations of the same sign. A group of metabolites was specifically highlighted in T22 plants, and included ADP, AMP, citric acid, dihydro-caffeic acid,2-hydroxyglutarate, phosphoenolpyruvate (PEP), coumarin, syringaldehyde and tetrahydrofolate being down-accumulated in T22 vs. CTRL and T22Aph vs. T22, and up-represented in Aph vs. CTRL; and Uroporphyrinogen III and Galactonate/Gluconate, showing an opposite trend. Interestingly, over-represented metabolites retrieved in T22 samples are precursors of salicylic acid: Salicylate β-D-glucose ester and Salicyloyl-L-aspartic acid, have a concentration three times higher than the control. Furthermore, Phenylalanine, Coumaric and chorismic acids, member of phenilpropanoids and known for their possible flow into primary steps of salicylic acid biosynthesis, in T22 samples are about 5 times higher than in control (Supplementary Table 6). Fourteen metabolites were found to be specifically associated with the presence of *T. harzianum* (T22 vs. CTRL and T22Aph vs. Aph comparisons), mostly with alterations of negative sign (indicated in Supplementary Table 6 with a cross). Finally, 7 DAMs each were exclusive for T22Aph vs. T22; among them, δ-tomatine (an alkaloid), N-isovalerylglycine and threonine/homoserine (amino acids) and protoporphyrinogen IX, an isoprenoid associated with tissue necrosis, were detected at lower levels over the control. At the opposite, the amino acid glutamic acid, the sugar phosphate glycerate-2-P/glycerate-3-Pand the lipid CDP-choline displayed a higher accumulation in T22Aph leaves over the CTRL. T22 and T22Aph were found to share 32 metabolites: interestingly, most of them varied differently between the two comparisons, with the exception of shikimic acid and dihydrokaempferol-7-O-glucoside, and raffinose/melezitose, respectively, down- and over-accumulated in both T22 and T22Aph. In the group of compounds showing higher accumulation in T22Aph, two relevant alkaloids were found (α-/β-tomatine), together with other compounds (indicated in Supplementary Table 6 with a hash mark). On the contrary, a group comprising the amide feruloylputrescine and other compounds (indicated in Supplementary Table 6 with an asterisk), resulted more abundant in T22 vs. CTRL than T22Aph vs. T22. Notably, no common compounds were found between T22Aph and Aph.

**Figure 8:**
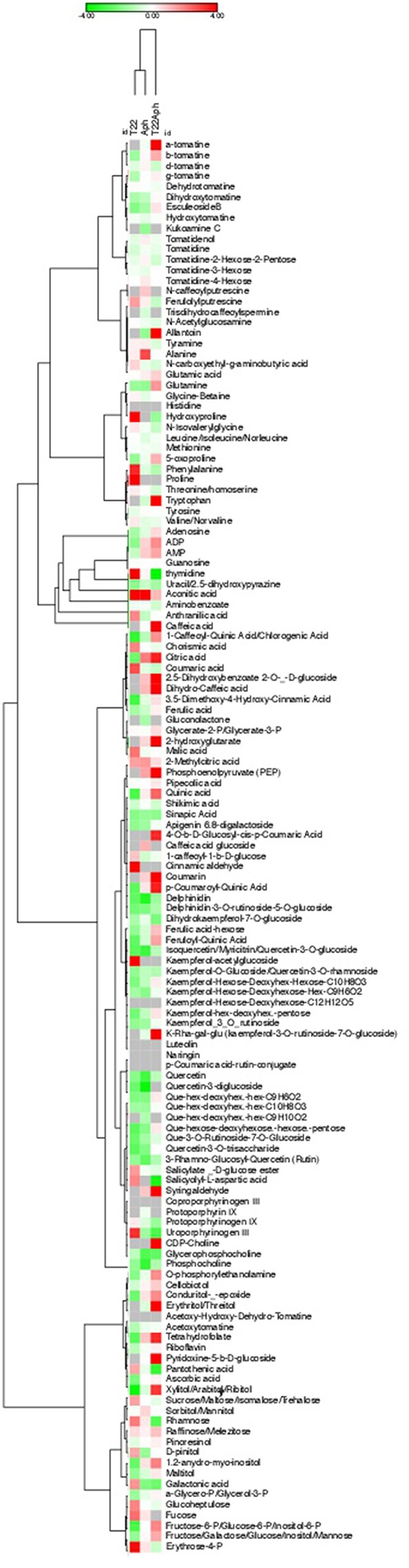
Hierarchical Clustering (HCL) of semi-polar metabolome of tomato leaves grown in the absence and in the presence of the aphid *M. euphorbiae* and the fungus *T. harzianum*, alone or in combination. Colored squares represent the values of log2-transformed fold changes of a metabolite with respect to the corresponding control (water control CTRL for Aph and T22 samples; T22 for T22Aph), according to the color scale shown (green: down-accumulated; red: up-accumulated). Gray squares indicate no detectable accumulation of the corresponding metabolite. Hierarchical clustering was calculated both on columns and rows, applying the One Minus Pearson correlation with the average linkage algorithm.

Targeted semi-polar metabolomes were used to generate a Hierarchical Clustering (HCL), applied both on columns and rows, in order to study the global relationships within leaves treated with *M. euphorbiae* and/or *T. harzianum*T22 (Figure 8). Interestingly, two distinct groups were produced, with one-to-one interactions (aphid or fungus, Aph vs. CTRL and T22 vs. CTRL) on the left side, and the three-way interactions (T22Aph vs. T22) clustering alone. As expected, metabolites displaying similar trends of related accumulation over the controls grouped together like, for instance, a set of alkaloids in the initial part and one of phenylpropanoids in the central parts of the HCL.

Finally, we exploited the untargeted metabolomes to retrieve new ions not included in our targeted database and specific for the four conditions under investigation (Supplementary Table 9). Twenty-two ions were found, which were subjected to metabolomics database interrogation, and isotopic pattern ratio, literature search and standard (where available) validations. For nine of them, an identification already reported in tomato was found: this group of metabolites included three acids (5-amino-levulinic acid, hydroxypipecolic acid and glutaric acid) and one phenylpropanoid (caffeic acid hexose II), over-accumulated in T22Aph vs. T22. In addition, one ester-like (2-Amino-2-methylbutanoate), two lipids (CDP-DG(16:0/20:4(5Z,8Z,11Z,14Z)), LysoPE [22:6(4Z,7Z,10Z,13Z,16Z,19Z)/0:0)], and one alkaloid (C33H57NO8 (Jurubine-like), showing an opposite trend. Thirteen additional molecular ions were detected, which could not be identified as any of the known tomato metabolites, and were tentatively assigned according other metabolite identifications: four of them (2 acids: 3-hydroxybutyric acid-like, citric acid-like; an amine: N-methyl ethanolamine phosphate-like; a flavonoid: 6-hydroxy-4′-methoxyflavone-like; and an alkaloid: O-acetylnitraraine-like) and 3 ions (a flavonoid: 3′,4′-dihydroxychalcone-like; an alkaloid: beta-obscurine-like; and an unknown) were found at, respectively, higher and lower levels in T22Aph vs. T22. Interestingly, most of the cited molecules displayed an inverse statistically significant accumulation in the other two comparisons (Aph vs. CTRL and T22Aph vs. T22).

## Discussion

Plants colonized by *Trichoderma* have often shown multiple beneficial effects (Hermosa et al., 2012; Vitti et al., 2015). We previously demonstrated an enhancement of the indirect defense barriers in tomato plants treated with *T. harzianum* T22, which were more attractive toward aphid parasitoids (Coppola et al., 2017a). This plant phenotype was associated with an increased level of methyl-salicylate and β-caryophyllene, known to be among the most active compounds in promoting *A. ervi* flight (Sasso et al., 2009; Coppola et al., 2017a).

Here we studied the impact of *T. harzianum* T22 colonization of tomato plant on direct defense responses to the aphid *M. euphorbiae*, and used transcriptomic and metabolomic approaches to shed light on the molecular mechanisms underlying the observed phenotypic changes.

### Aphid Infestation Suppresses Plant Defense Responses

Aphid feeding on the tomato cv “Dwarf San Marzano” induced dramatic changes in thetranscriptome and metabolome of the plant, despite the very limited mechanical damage caused by the insect. Transcriptomic reprogramming was characterized by the deregulation of a large number of transcripts, the majority of which are down-regulated. The total number of DEGs was much higher than previously observed (Avila et al., 2012; Coppola et al., 2013), possibly due to the higher sensitivity of the digital method (RNA-Seq with respect to the analog one (microarray and/or the different tomato cultivars used in the two studies. However, the related GO categories distribution was in general agreement with previous reports Avila et al., 2012; Coppola et al., 2013. All levels of defense responses were influenced in Aph samples: oxidative stress, signal transduction, TFs and late defenses. The down-regulation of key-genes of plant immunity such as MAP Kinases, WRKY and genes associated with direct (i.e., protease inhibitors, PIs) and indirect (i.e., sesquiterpene synthase 1) responses, is consistent with the aphid capacity to circumvent host defenses by secreting evolutionarily conserved effectors able to suppress plant immune responses (Will et al., 2007; Elzinga et al., 2014). Aphid ability to interfere with plant defense mechanisms is clearly evidenced by the down regulation of genes associated with sugar metabolism and amino acid biosynthesis. Fructose-1, 6-bisphosphate aldolase is a key-enzyme involved in glycolysis, gluconeogenesis, and the Calvin cycle. It plays significant roles in biotic and abiotic stress responses, as well as in regulating growth and development processes (Lv et al., 2017). Sucrose synthase is a glycosyltransferase enzyme that plays a key-role in sugar metabolism. Sucrose is engaged in plant defense by activating plant immune responses against pathogens (Tauzin and Giardina, 2014). Threonine deaminase is part of the phytochemical arsenal that plants use to deter herbivores. Together with PIs and other defense-related compounds, is tightly regulated by the JA signaling pathway (Chen et al., 2005). The enzyme acts in the insect gut to degrade the essential amino acids arginine and threonine, respectively. In aphids it was observed that a shortfall in threonine contribute to the poor performance of the *Aphis fabae* on *Lamium purpureum* (Wilkinson et al., 2001). Tryptophan biosynthesis and the enzymes involved are induced by a wealth of stress agents, such as for instance, ozone (Conklin and Last, 1995) and biotic stress (Brader et al., 2001). In addition, a strong down-regulation of transcripts encoding Proteinase inhibitors (PIs) and several other glycosyltransferases further demonstrated the aphid ability to repress plant defense responses. PIs are proteins involved in defense responses and are often induced upon attack by insect herbivores, as they are able to inhibit insect growth and survival by disrupting their digestive physiology (Ryan, 1990; Lawrence and Koundal, 2002; Zhu-Salzman and Zeng, 2015). In aphids, similarly to what observed in thrips, PIs may inhibit aphid salivary proteases during probing and feeding establishment (Pyati et al., 2011; van Bel and Will, 2016) reducing the insect ability to degrade sieve-tube sap that includes proteins involved in defense (Furch et al., 2015). It was proposed that plant protect sap-proteins degradation by glycosylation that appears to prevent proteolysis (Taoka et al., 2007; Russel et al., 2009). Considering that glycosyltransferases are enzymes that catalyse the transfer of a sugar residue from an activated donor to an acceptor molecule the concerted down-regulation of transcripts encoding PIs and glycosyltransferases in Aph plants could be part of the aphid strategies to reduce the effectiveness of plant defense. Interestingly, three down-regulated glycosyltransferases (Solyc10g084890.2, Solyc03g078780.2, Solyc10g085280.1) showed high homology to UGT76B, C and/or E enzymes, which in Arabidopsis are involved in flavonoid biosynthesis and/or defense responses (Yonekura-Sakakibara and Hanada, 2011). The former function is consistent with the reduction in flavonoids, particularly kaempferol and quercetin glucosides, as well in phenolic acid derivatives, observed in aphid-infested plants (Supplementary Table 6).

Other down-regulated transcripts associated with plant defense are those encoding sesquiterpene synthase 1, Z,Z-farnesyl pyrophosphate synthase and geranylgeranyl reductase (Dudareva et al., 2004; Schmidt et al., 2011). Terpenoids, including sesquiterpenes and diterpenes, constitute some of the commonly encountered chemical classes of phytoalexins, biochemicals that locally protect plant tissues (Li et al., 2015). They are pathogen- and insect-inducible, known for their role in the attraction of predators, parasitoids, and other natural antagonists (Aljbory and Chen, 2018).

Among down-regulated genes, transcripts involved in phenylalanine metabolism (PAL) were retrieved, indicating a strong perturbation in phenylpropanoid pathway. In fact, as shown by the KEGG analysis, down-regulated genes involved in phenylalanine metabolism are in the early steps of the pathway, allowing the hypothesis of a possible accumulation of phenylalanine, that has been underlined as a crucial channel of SA biosynthesis (Chen et al., 2009). This finding is consistent with the reduced accumulation of several metabolites belonging to phenylpropanoid family in Aph plants that are located downstream PAL in the pathway. The over-presence of caffeic acid glucoside and coumarin could be similarly interpreted: the effect of the partial suppression of a branch of the phenylpropanoid biosynthesis causes the accumulation of central metabolites that are not toxic *per se* for aphids, but are precursor of molecules toxic for other herbivores (Sun et al., 2016).

### *Trichoderma harzianum* T22 Boosts the Plant Immune Response

The advantages conferred to the plant by *Trichoderma* were largely associated with biological control of phytopathogens (Woo et al., 2006; Lorito et al., 2010). However, in the past 30 years, particularly with the advancement of modern techniques to analyse plant-microbe interactions, it became increasingly evident that root colonization by *Trichoderma* is associated with a wealth of beneficial effects, by activating defense responses against multiple stressors (De Meyer et al., 1998; Yedidia et al., 1999; Harman et al., 2004; Lorito et al., 2010; Shoresh et al., 2010; Hermosa et al., 2012; Lorito and Woo, 2015; Manganiello et al., 2018). Regarding plant responses to phytopathogens, the production of microbe-associated molecular patterns (MAMPs) by *Trichoderma* enhances the sensitivity of first defense, by maintaining a level of “alert” near to the threshold of effective resistance (Lorito et al., 2010). In particular, *Trichoderma* is also known to be involved in priming, the activation of plant defense prior to invasion, whereby upon pathogen attack *Trichoderma* stimulates a faster response to the pathogen effectors or it produces compounds specifically recognized by plant receptors able to elicit defense mechanisms (Lorito et al., 2010; Mauch-Mani et al., 2017; Manganiello et al., 2018). Only very recently, this priming response was also proposed to have a role in tomato indirect defense against aphid (Balmer et al., 2015; Coppola et al., 2017a; Tan et al., 2017). Here, we observed the activation of early signals of defense responses against insects in T22 plants that indicates the ability of these plants to mount more rapid and effective direct and indirect defense responses. Similarly, the up-regulation of transcripts coding for several types of TF is a peculiar feature of adaptive plant strategies that improve their defensive potential (Khong et al., 2008; Walling, 2008). On the other hand, the concurrent down-regulation of some defense-related functions observed in T22 plants is possibly due, at least in part, to fungal effectors that allow *T. harzianum* T22 to colonize plant roots as an avirulent symbiont (Shoresh et al., 2005).

*Trichoderma*, as many beneficial plant growth promoting rhizobacteria (PGPR), tends to activate induced systemic resistance (ISR) that involve signal transduction pathways responding to JA/ET, but includes also cross-talk with SA, as well as with phytohormones associated with plant development (Harman et al., 2004; Shoresh et al., 2005; Hermosa et al., 2012). A trade-off is established between plant biosynthetic pathways involving defense or cellular/growth functions that can be regulated by *Trichoderma* stimuli, such as 1-aminocyclopropane-1-carboxylic acid deaminase (ACCD) activity, that modulates ET biosynthesis, or indole-3-acetic acid (IAA), that stimulates plant growth (Pieterse et al., 2009; Hermosa et al., 2012).

Our findings indicates that fungal colonization of tomato has an impact on phosphorylation dynamics of several Serine/threonine- and Leucine-rich repeat protein kinases, that were up-regulated. These kinases are involved in recruiting signals from receptors sensing environmental conditions and phytohormones and recalibrating them into appropriate outputs such as changes in metabolism, and gene expression, to activate defense/resistance against invaders (Xu and Huang, 2017). This evidence supports the hypothesis that *Trichoderma* T22 strain triggers a “defense mood” in the tomato cultivar “Dwarf San Marzano,” generating a pre-alerted state of “priming” to face more efficiently likely incoming attacks (Conrath, 2011; Conrath et al., 2015). However, it is of interest to note that this reinforcement of defense barriers is not univocally associated with *Trichoderma* infection of tomato plants. Indeed, what observed here in terms of direct defense for SM was quite different in the case of *Trichoderma longibrachiatum* strain MK1, which similarly increased plant attractiveness toward the aphid parasitoid *A. ervi*, but also promoted the development and reproduction of *M. euphorbiae* (Battaglia et al., 2013). This demonstrates that the plant response can be different to different fungal species, and can be specific for each tomato variety, as already suggested by Tucci et al. (2011).

The up-regulation of a Multicystatine and several other Proteinase inhibitors with the T22 treatments (observed in both T22 and T22Aph) correlated with the reduced aphid survivorship overtime. This plant defense barrier induced by T22 was reinforced by the concurrent reduction in the number of down-regulated transcripts by aphid feeding related to other protease inhibitors (4 in Aph and 2 in T22Aph), which further contributes to the disruption of the aphid-induced suppression of plant defense. Previous studies have demonstrated that *Trichoderma* interferes with nematode performance by inducing Protease inhibitors in tomato (Martínez-Medina et al., 2017). In addition, in wheat the fungus counteracts nematode growth, inducing chitinase, β-1, 3-glucanase and defense compounds such as total flavonoids and lignin (Zhang et al., 2017). The metabolomics analysis remarkably expands the understanding of effect induced by *T. harzianum* T22 on tomato defenses when coupled with insect feeding. Defense-related secondary metabolites were over-represented in T22Aph samples compared to T22 or with only aphid infestation (Aph). The defense barrier array involved alkaloids (α-/β-tomatine) that could be responsible for the reduction in aphid survival together with late defense gene products (PPO, LapA, Miraculin, and many others), phenolic acids and flavonoids.

**Figure 9:**
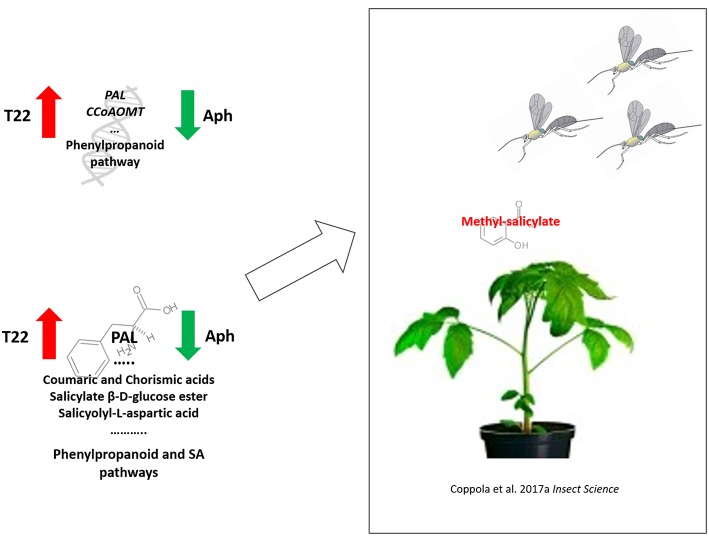
Proposed summary of transcriptomic and metabolomics changes imposed by *T. harzianum* T22 colonization on tomato plants responsible of the promoted direct and indirect defense responses against aphids. Starting from the left side, phenylpropanoid, and SA pathways cover a central role in T22-induced defenses at transcriptional (up) and metabolomics (down) levels. Promoted pathways are the source of MeSA, a volatile compound responsible of the increased attractiveness toward the parasitoid wasp *A. ervi*.

The up-regulation of the enzymes participating at different stages in the phenylpropanoid biosynthesis, for example, involved in catalyzing key-steps such as the conversion of phenylalanine in cinnamic acid (Phenylalanine ammonia-lyase and Caffeoyl-CoA O-methyl transferase), or catalyzing final branches for lignin production (i.e., Peroxiredoxin), may be associated with the observed increased level of compounds implicated in the defense responses. In fact, phenylpropanoids or their precursors/derivates may exert direct toxicity against insect herbivores (Naoumkina et al., 2010) and, at the same time, are precursors to VOCs that contribute to plant indirect defense (Dudareva et al., 2013). Notably, *PAL* is the up-regulated gene with the highest fold change in T22 plants while it is down-regulated in aphid-infested plants (Aph). The observed transcriptomic reprogramming of phenylpropanoid pathway is consistent with the augmented accumulation of Phenylalanine, Coumaric and Chorismic acids, Salicylic acid precursors, as well as SA-related metabolites (Salicylate β-D-glucose ester and salicyolyl-L-aspartic acid) in T22 plants. These observations are summarized in Figure 9. Similarly, metabolites involved in PAL pathway were over-accumulated in T22 plants. This is consistent with the previously observed increased attractiveness toward *A. ervi* mediated by methyl-salicylate (Coppola et al., 2017a).

Furthermore, T22 plants showed higher accumulation of sugars quantities than CTRL plants, indicating a higher root uptake and photosynthesis efficiency, as confirmed by the over-representation of a series of transcripts of the Calvin cycle; this is in line with the reported beneficial effect of the fungus on the plant physiology (Lorito et al., 2010; Lorito and Woo, 2015) and consistent with previous observation on *Trichoderma* –tomato interaction (De Palma et al., 2019).

The higher content of sugar could be the result of the over-expression of a large group of genes associated with cellular and metabolic processes and many others identified by GO annotation (Figure 3; Supplementary Table 2). These processes produce substances such as nutrients, hormones, metabolites that contribute to the positive effects observed in plant growth promotion frequently induced by *Trichoderma* spp. (Tucci et al., 2011; Vinale et al., 2014; Lorito and Woo, 2015). It has been proposed that plant-derived sugars represent not only a carbon source for the fungus, but also a tool to modulate the extension of root colonization and the systemic induction of photosynthesis in leaves (Vargas et al., 2009). In addition, the increased expression of glycolytic enzymes can redirect the higher sugar flux to increase the carbon supply to biosynthetic pathways involved in the production of plant resistance-secondary metabolites (Fürstenberg-Hägg et al., 2013). In agreement with this hypothesis, a large group of terpenes and carotenoids/apocarotenoids genes were up-regulated in T22 leaves. In the same context, amino acid metabolism was strongly affected by *Trichoderma* and aphids: among the different transcript/metabolite data, a group of elements involved in glutamate metabolism was highlighted. For instance, a*sparagine synthetase 1* and *aspartate aminotransferase*, involved in glutamate production, were up-regulated in T22Aph; interestingly, a plant resistance mechanism due to increased levels of glutamate has been proved (Dixit et al., 2013). A possible explanation for this finding, besides its role in chlorophyll pathway (see below), could rely on the involvement of glutamate in tricarboxylic acidreplenishment and nitrogen remobilization upon insect attack (Ameye et al., 2018). In agreement with transcriptomic data, glutamate accumulation was observed in T22Aph vs. T22 leaves. In the same context, additional genes of glutamate synthesis/sensing/catabolism varied according the same trend: *proline dehydrogenase*, converting proline in Δ1-pyrroline-5-carboxylate, and which is known to contribute to the hypersensitive response and disease resistance (Cecchini et al., 2011), were up-regulated; similarly, *glutamate receptor 1.2*, for which a potential function as primary sensors in plant defense responses has been postulated (Forde and Roberts, 2014), displayed positive changes.On the contrary, *glutamate decarboxylase*, opposing glutamate accumulation by its conversion in γ-aminobutyrate, was down-expressed in T22Aph over CTRL leaves.

### Key Events of the Tripartite Interaction

The hormonal balance during tomato-aphid interaction in presence of *T. harzianum* T22 is very delicate, variable and complex. *Trichoderma* spp. induction of ethylene and jasmonate (ET/JA) and salicylic acid (SA)-mediated signaling pathways has been reported in tomato cv. MicroTom (Manganiello et al., 2018). In our dataset, specific genes of the tripartite interaction are involved in ethylene biosynthesis and signaling, confirming the impact of *Trichoderma* on this pathway. ET production is part of the array of defense responses triggered in different plants by aphid feeding (Mantelin et al., 2009; Coppola et al., 2013). Indeed, the tomato *ERF Pti5* gene confers protection against aphids, both in susceptible and resistant genotypes (War et al., 2015).

The host plant regulation by hormonal management exerted by *T. harzianum* T22 in the tripartite interaction is also based on the down-regulation of several members of WRKY family of transcription factors, known for their promotion of JA signaling through the negative interplay with SA pathway (Li et al., 2004; Takatsuji, 2014). WRKYs represent keystones of communication between JA and SA and are involved in multiple defense responses (Phukan et al., 2016). These TFs appear to be aphid targets in the manipulation of plant host resistance (Kloth et al., 2016). Their down-regulation in T22Aph plants promotes JA-mediated defenses, at the expenses of SA signaling which would interfere with *Trichoderma* colonization of plant roots. In other words, the aphid strategy based on the activation of the salycilate pathway to exert a negative regulation of JA signaling, to which they are sensitive, is outcompeted by the capacity of the Trichoderma strainT22 to counteract it, as it is is detrimental for the fungal entry and development in the plant tissues. Many TFs implicated in JA signaling have been identified and functionally characterized, including many basic helix–loop–helix (bHLH) type TFs (Zhou and Memelink, 2016). To date, four subclades of the bHLH TF family have been implicated in JA signaling in *Arabidopsis*, each with a different contribution to the JA response (Goossens et al., 2017). The redirection of the expression of a bHLH TF, up-regulated in T22 while down-regulated in Aph plants, represents a further contribute of *Trichoderma* colonization to defense priming against aphids and, possibly, other herbivores. Notably, allantoin, a purine metabolite that activates JA signaling in *Arabidopsis thaliana* (Takagi et al., 2016), was found to accumulate at higher levels in T22Aph vs. T22 (Supplementary Tables 6, 7). In addition, a gene encoding a steroid dehydrogenase, (upregulated in T22Aph and down regulated in Aph) is involved in plant responses to stress through lipid signaling (Fürstenberg-Hägg et al., 2013). Membrane lipids serve as substrates for the generation of numerous signaling lipids such as phosphatidic acid, phosphoinositides, sphingolipids, lysophospholipids, oxylipins, N-acylethanolamines, free fatty acids and others. These molecules are tightly regulated and can be rapidly activated upon abiotic stress signals (Hou et al., 2016) or pathogen attack (Okazaki and Saito, 2014). Interestingly, O-phosphorylethanolamine, an intermediate of ethanolamine/choline synthesis, which can take part in the stress response-signaling machinery, was over-accumulated in T22Aph leaves (Supplementary Tables 6, 7). Phloem lipids have been associated not only with intracellular signaling but also with a long-distance lipid signaling: lipid molecules could be released upon a stress perception and moving through the phloem they could bind receptors with the consequent modification of the sink tissue mediating a response (Benning et al., 2012). In this scenario, the possible alteration of lipid signaling following *T. harzianum* T22 colonization of tomato roots could contribute to tomato responses in the initial phase of perception and recognition of the injury.

Interestingly, among the metabolites identified by targeted/untargeted metabolomics, and previously reported in tomato, 5-amino-levulinic acid, while reduced in Aph, is highly overproduced in T22 and in T22Aph plants. This metabolite is known to be effective in counteracting the damages of different plant stressors (Yang et al., 2014). Similar accumulation pattern was registered for hydroxypipecolicacid, very recently identified as a mobile signal responsible of the induction of systemic disease resistance in Arabidopsis (Chen et al., 2018); chlorogenic and sinapic acids, which can improve host plants resistance (Nićiforović and Abramovič, 2013; Kundu and Vadassery, 2019); anthranilic acid, precursor of methyl anthranilate, which has been associated with the production of the volatile blend attracting herbivore parasitoids (Köllner et al., 2010).

Concerted regulation of genes and metabolites involved in chlorophyll metabolism is observed in the plant-fungus-insect interaction. Aphids repress, and *Trichoderma* induces, two early intermediates in chlorophyll biosynthesis; 5-amino-levulinic acid (ALA) and uroporphyrinogen III (UROIII); later intermediates (coproporphyrinogenIII (COPIII), protoporphyrin IX (PPIX)) show the opposite trend (Supplementary Table 10). This dual regulation is observed also for transcripts involved in chlorophyll biosynthesis: for instance, coproporphyrinogen III oxidase (CPOX) is induced by aphids, while transcripts encoding later steps (magnesium chelatase H subunit (MgCH), magnesium-protoporphyrin monomethyl ester cyclase (MPEC)) and a light harvesting chlorophyll a/b binding protein (LHC) are repressed. *Trichoderma* induces uroporphyrinogen decarboxylase (UROD) and protochlorophyllide reductase (POR) as well as MgCH, MPEC and two LHCs. Finally, in the triple interaction, aphid infestation seems to be epistatic over *Trichoderma* treatment, since it represses ALA and UROIII, strongly induces a fifth intermediate (protoporphyrinogen IX) and represses a series of transcripts involved in chlorophyll biosynthesis and sequestration (MgCH, PEC and 23 LHCs). Such a coordinated regulation of transcripts and metabolites belonging to a single pathway must have a biological meaning. Several genes in the chlorophyll pathway are known to be involved in plant defense responses: for instance, a null *UROD* mutation generates a disease lesion mimic phenotype in maize (Hu et al., 1998), and the *accelerated cell death 2* gene of *Arabidopsis*, showing constitutive activation of defenses in the absence of pathogen infection, encodes a red chlorophyll catabolite reductase (Mach et al., 2001). Two hypotheses have been proposed explaining the remodeling of chorophyll metabolism in defense responses: in the first hypothesis, a reduction in chlorophyll biosynthesis and accumulation in aphid-resistant cultivars would cause a decrease in photosynthetic efficiency, thus limiting the nutrient supply to aphids (Carrillo et al., 2014). This hypothesis is consistent with the data from the tripartite (plant-fungus-aphid) interaction. A second hypothesis is based on the well-known role of some tetrapyrrole chlorophyll precursors in generating Reactive Oxygen Species in the presence of light, which in turn activate defense responses. This hypothesis is consistent with some, but not other, data presented in this paper: for instance, both UROD and CPOX silencing causes the accumulation of photosensitizing tetrapyrroles and necrotic lesions in tobacco (Mock et al., 1998). In the tomato-Trichoderma-aphid system, UROD is induced by Trichoderma (presumably a way to alleviate the production of ROS by reducing the levels of photosensitizing COPIII and PPIX) while CPOX is induced by aphid infestation (presumably a way to reduce the levels of COPIII). It is not entirely clear from our data whether the interplay of these two responses enhances or diminishes resistance to aphids in the triple interaction.

In conclusion, our study suggests a wide, articulated and sophisticated contribute of *T. harzianum* T22 in the promotion of tomato endogenous defenses against phloem-feeders, by the instauration of a preparation to defense. This preparation ranges from transcriptomic to metabolomics changes, from early signals to late effector of defense responses. In the specific tripartite system under investigation, the major contribute of the beneficial fungus appears to be the manipulation of phloem sap sentinel molecules, the regulation of hormonal balance and enhanced communication with natural enemies via terpenes and salycilate.

## Data Availability

Publicly available datasets were analyzed in this study. This data can be found here: https://www.ncbi.nlm.nih.gov/Traces/study/?acc=PRJNA532377.

## Ethics Statement

This article does not contain any studies with human participants or animals performed by any of the authors.

## Author Contributions

MC performed and analyzed transcriptomic data and draft the ms. GD and GG performed metabolomics, analyzed the results and contributed to manuscript writing. MD performed insect bioassays and analyzed the data. SW participated to the experimental design and contributed to manuscript writing. DM contributed to RNA isolation. ML contributed to the work plan and helped in results interpretation. FP contributed to the work plan and to data analyses and revised the manuscript. RR conceived and designed the study, supervised the experimental work and wrote the paper.

### Conflict of Interest Statement

The authors declare that the research was conducted in the absence of any commercial or financial relationships that could be construed as a potential conflict of interest.

## Supplementary Material

Supplementary Figure 1 GO categories distribution for plants treated with *T. harzianum* T22 in the “Biological Process” domain. Colors indicate the enrichment score of each GO category for **(A)** up-regulated and **(B)** down-regulated genes.

Supplementary Figure 2 GO categories distribution for SM plants infested by *M. euphorbiae* for 48 h in the “Biological Process” domain. Colors indicate the enrichment score of each GO category for **(A)** up-regulated and **(B)** down-regulated genes.

Supplementary Figure 3 GO categories distribution for SM plants treated with *T. harzianum* T22 and subsequently infested by aphids in the “Biological Process” domain. Colors indicate the enrichment score of each GO category for **(A)** up-regulated and **(B)** down-regulated genes.

Supplementary Figure 4 Expression analysis of selected defense genes from the tomato DEGs by Real Time RT-PCR in plants: treated with *T. harzianum* T22 (T22); infested by aphid *M. euphorbiae* (Aph) or treated with T22 and subsequently infested by aphid (T22Aph). Relative quantities (RQ) are calibrated to untreated plants (Ctrl), as indicated by the linear scale on the Y-axis. Asterisks indicate statistically significant differences compared to control condition (^*^*p* < 0.05; ^**^*p* < 0.01; ^***^*p* < 0.001 *t*-test).

Supplementary Figure 5 Principal Component Analysis (PCA) of the untargeted metabolomic analysis, of the semi-polar fraction, obtained from tomato leaves grown in the absence and in the presence of the aphid *M. euphorbiae*, and treated with the *T. harzianum* T22, alone or in combination with the aphids. Dots with same colors indicate biological experimental replicates. For more details, see Materials and Methods.

Supplementary Figure 6 Heatmap (HM) of semi-polar metabolome of tomato leaves grown in the absence and in the presence of the aphid M. euphorbiae and the fungus *T. harzianum* T22, alone or in combination. Colored squares represent the values of log2-transformed fold changes of a metabolite in respect to the corresponding control (water control CTRL for Aph and T22 samples; T22 for T22Aph), according to the color scale shown (green: down-accumulated; red: up-accumulated). Gray squares indicate no detectable accumulation of the corresponding metabolite.
