# Supplementary figures and images for: Transcriptome and Metabolome Reprogramming in Tomato Plants by Trichoderma harzianum strain T22 Primes and Enhances Defense Responses Against Aphids
Source: Front Physiol. 2019 Jun 21;10:745. doi: 10.3389/fphys.2019.00745 (PMC6599157; doi:10.3389/fphys.2019.00745)

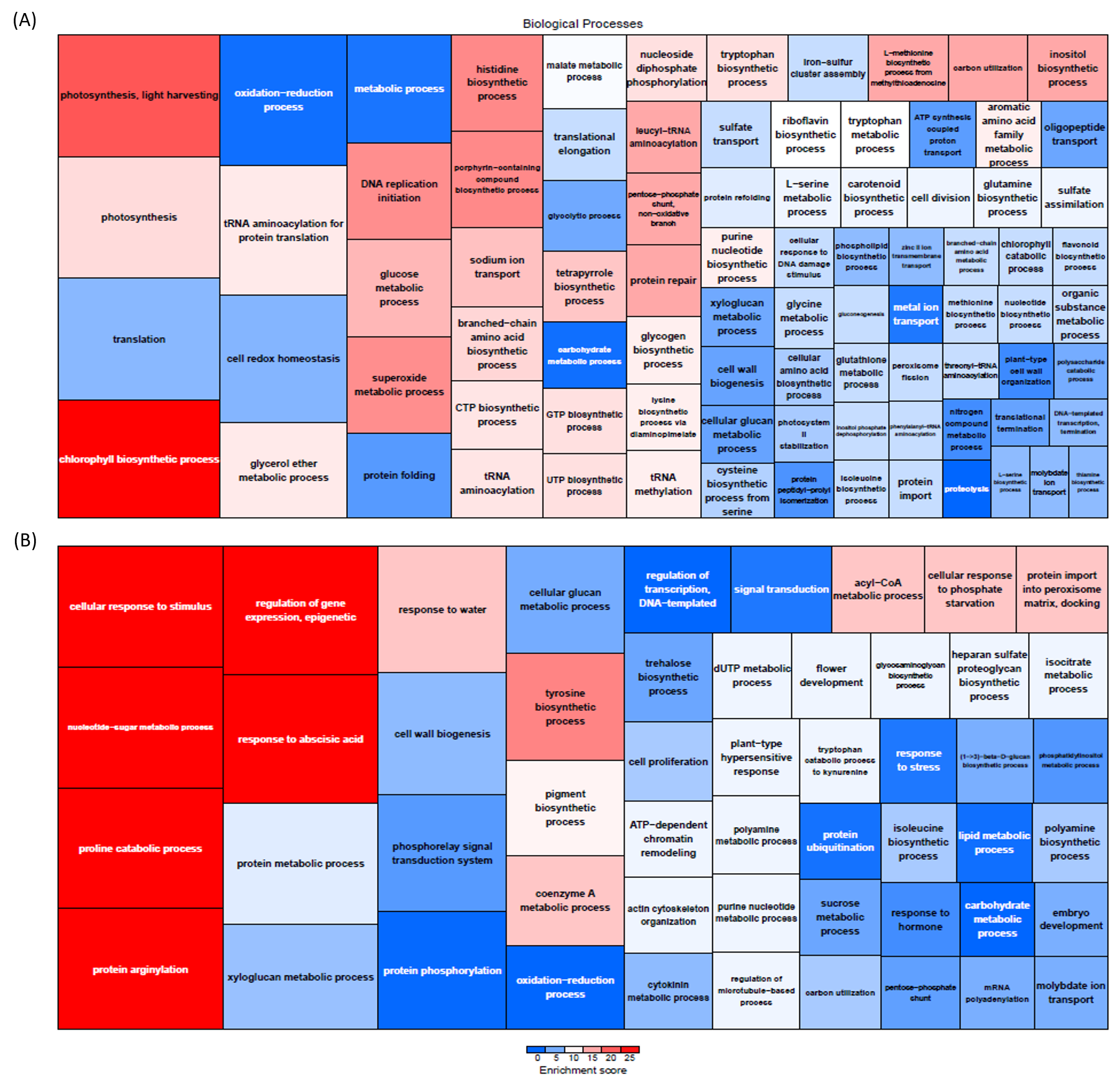

Supplement: Supplementary Figure 1 — GO categories distribution for plants treated with T. harzianum T22 in the “Biological Process” domain. Colors indicate the enrichment score of each GO category for (A) up-regulated and (B) down-regulated genes. [file Image_1.TIF]

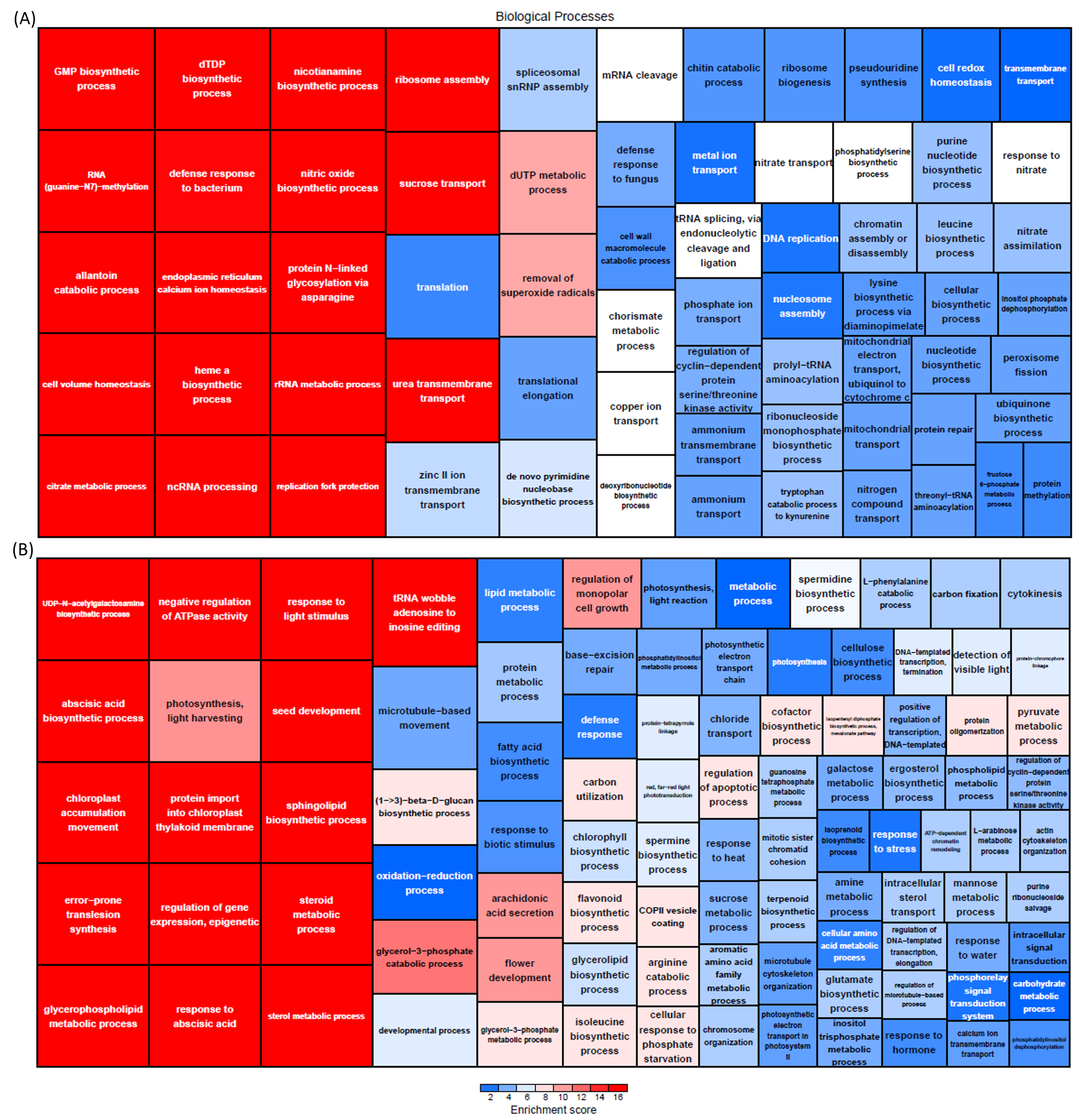

Supplement: Supplementary Figure 2 — GO categories distribution for SM plants infested by M. euphorbiae for 48 h in the “Biological Process” domain. Colors indicate the enrichment score of each GO category for (A) up-regulated and (B) down-regulated genes. [file Image_2.TIF]

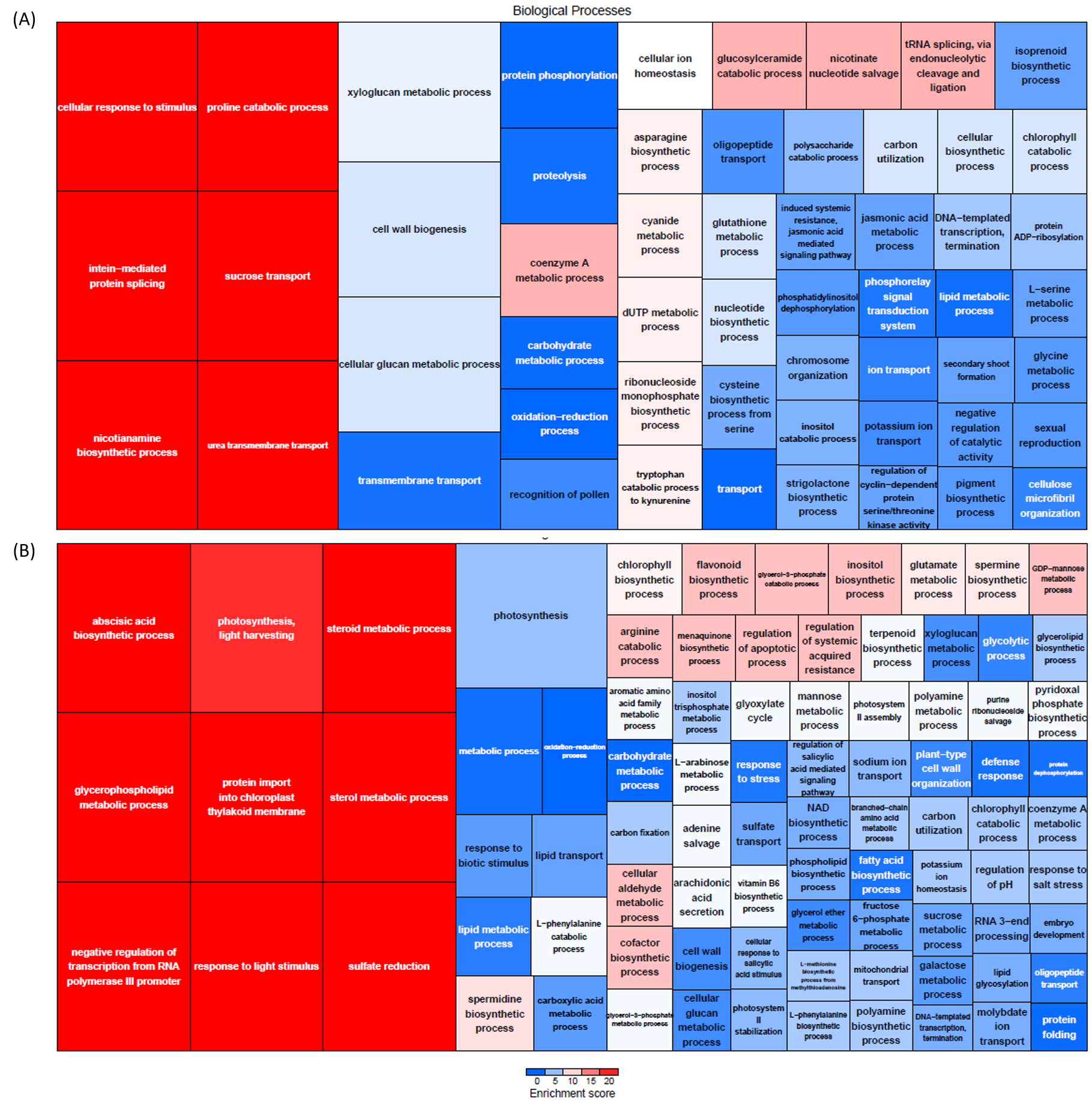

Supplement: Supplementary Figure 3 — GO categories distribution for SM plants treated with T. harzianum T22 and subsequently infested by aphids in the “Biological Process” domain. Colors indicate the enrichment score of each GO category for (A) up-regulated and (B) down-regulated genes. [file Image_3.TIF]

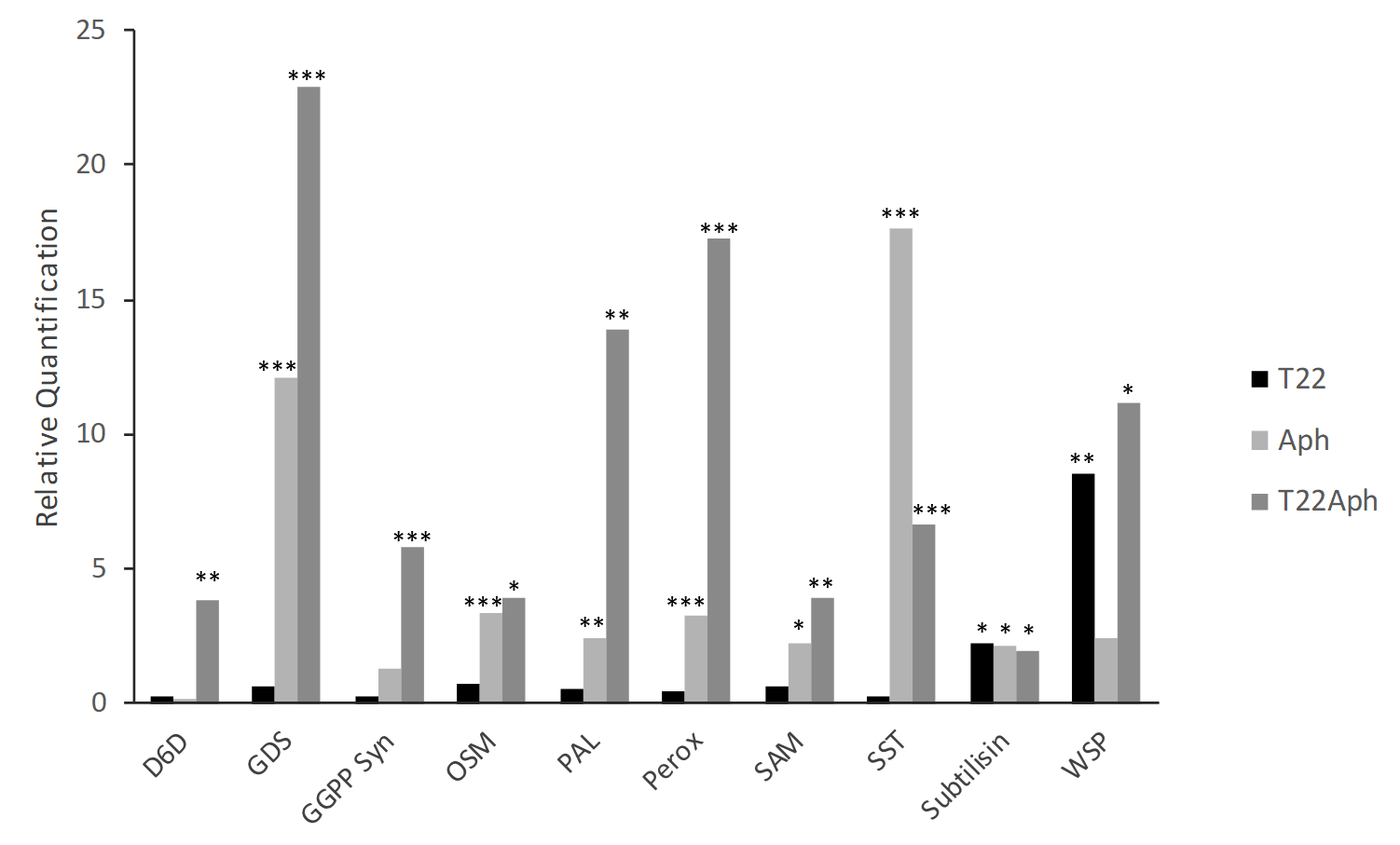

Supplement: Supplementary Figure 4 — Expression analysis of selected defense genes from the tomato DEGs by Real Time RT-PCR in plants: treated with T. harzianum T22 (T22); infested by aphid M. euphorbiae (Aph) or treated with T22 and subsequently infested by aphid (T22Aph). Relative quantities (RQ) are calibrated to untreated plants (Ctrl), as indicated by the linear scale on the Y-axis. Asterisks indicate statistically significant differences compared to control condition (*p < 0.05; **p < 0.01; ***p < 0.001 t-test). [file Image_4.TIF]

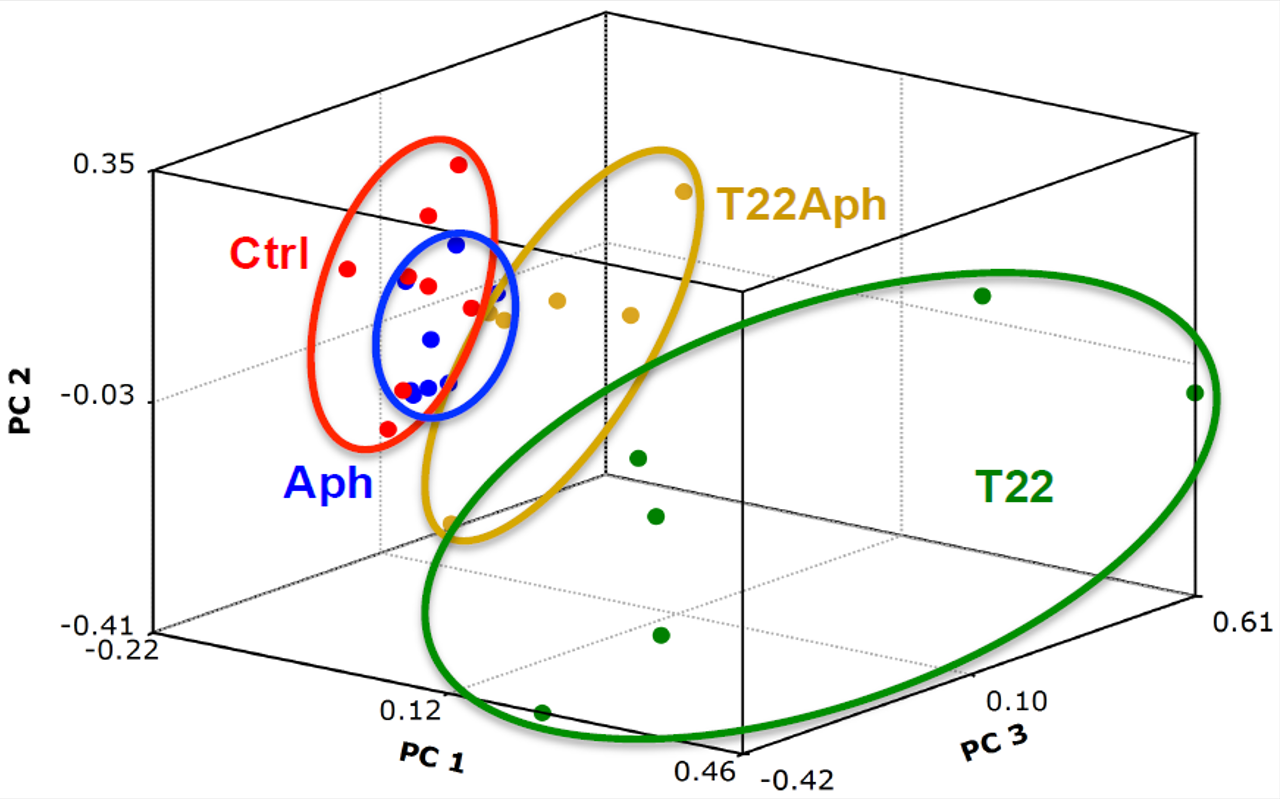

Supplement: Supplementary Figure 5 — Principal Component Analysis (PCA) of the untargeted metabolomic analysis, of the semi-polar fraction, obtained from tomato leaves grown in the absence and in the presence of the aphid M. euphorbiae, and treated with the T. harzianum T22, alone or in combination with the aphids. Dots with same colors indicate biological experimental replicates. For more details, see Materials and Methods. [file Image_5.TIF]

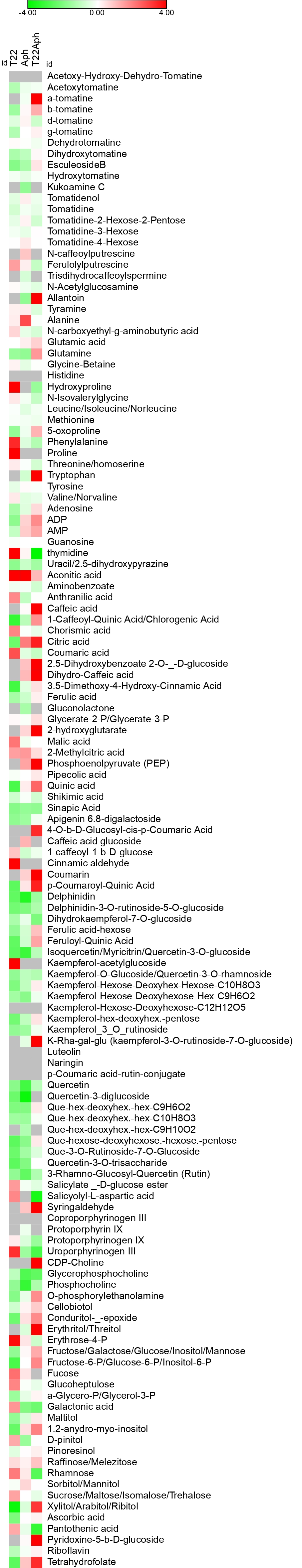

Supplement: Supplementary Figure 6 — Heatmap (HM) of semi-polar metabolome of tomato leaves grown in the absence and in the presence of the aphid M. euphorbiae and the fungus T. harzianum T22, alone or in combination. Colored squares represent the values of log2-transformed fold changes of a metabolite in respect to the corresponding control (water control CTRL for Aph and T22 samples; T22 for T22Aph), according to the color scale shown (green: down-accumulated; red: up-accumulated). Gray squares indicate no detectable accumulation of the corresponding metabolite. [file Image_6.JPEG]
